# A Dendrite‐Resistant Sodium/Porous‐Carbon Anode for Solid‐State Batteries: Strategies and Challenges for Low‐Pressure Operation

**DOI:** 10.1002/cssc.70879

**Published:** 2026-07-15

**Authors:** J. Mark Weller, Joseph P. Quinn, Evgueni Polikarpov, Henry Hyungkyu Han, Marcos Lucero, Guosheng Li

**Affiliations:** ^1^ Energy and Environment Directorate Pacific Northwest National Laboratory Richland Washington USA

**Keywords:** cryogenic ion‐milling, dendrites, low‐pressure, sodium beta alumina, solid‐state sodium battery

## Abstract

Sodium solid‐state batteries (Na‐SSBs) are attracting growing interest due to the abundance of Na over Li, but they still tend to fail under practical current densities and cycling capacities due to dendrites, and are therefore frequently evaluated under impractically high stack pressures. Here, a porous carbon interfacial layer is utilized in conjunction with Na‐ß″‐Al_2_O_3_ solid electrolytes (BASE) to enable Na‐cycling at milder cell pressures. This sodium/porous carbon layer enables improved solid‐state Na cycling in symmetric cells, up to a current density of 10 mA cm^−2^ at 25 °C. 1 mAh cm^−2^ capacity can be reliably cycled at 1 mA cm^−2^ at an elevated temperature of 60 °C in symmetric cells. Interfacial evolution is investigated via cryogenic ion milling and cross‐sectional imaging, revealing void formation, Na extraction from porous carbon, and/or delamination of the porous carbon matrix at the Na‐metal/BASE interface, depending on temperature, pressure, current density, and areal capacity. Despite these interfacial changes, excellent dendrite resistance is maintained. A quasi‐solid‐state full cell using a Na‐transition‐metal‐oxide cathode delivers an areal capacity of ∼2.7 mAh cm^−2^ at 0.125 mA cm^−2^. This work demonstrates an alternative pathway toward an Na‐metal anode in Na‐SSBs without excessive stack pressure.

## Graphical abstract

Sodiophilic mesoporous carbon confers a near‐perfect interface between Na metal and ceramic solid electrolytes, while serving as a host for solid Na anodes. This synergy forms a foundational element for sodium solid‐state batteries (Na‐SSBs) in pairing a dendrite‐resistant anode with an inherently (electro)chemically stable ceramic electrolyte. This foundation can be paired with a variety of cathode systems to meet diverse stationary energy storage demands.

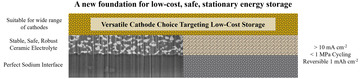

## 1 Introduction

For many years, solid‐state batteries (SSBs) have been viewed as the safest approach to utilizing high‐capacity alkali‐metal anodes for use in secondary batteries [1]. For example, dendrite formation in rechargeable Li or Na‐metal batteries can result in a short circuit, followed by thermal runaway and catastrophic failures [2, 3]. Although SSBs utilize a nonflammable solid‐state electrolyte (SSE), which most likely eliminates or reduces the risk of such extreme events, it has been reported that alkali‐metal dendrites can still intrude into the SSE and cause cell failure, often at impractically low current densities (e.g., <1 mA cm^−2^) [1]. While many factors contribute to low critical current densities (CCDs), including poor interfacial contact/wetting of the alkali metal and resistive interphases [4, 5].

In general, solid‐state diffusion of the alkali metal (Li, Na, etc.) anode is somewhat low at room temperature, though Na‐metal self‐diffusion is intrinsically higher than Li due to its lower melting temperature (97.8 °C for Na vs. 180.5 °C for Li metal) [6]. As a consequence, during higher current density Na‐metal stripping (*discharge*), voids tend to form at the Na‐metal/SSE interface as the rate of oxidation of Na and intercalation into the SSE outpaces the inherent Na‐diffusion rate in the metal anode [7]. This, in turn, results in increasing overpotential due to the reduced active surface area and focusing of current density onto the reduced contact area between Na and the SSE. On subsequent plating of Na (*charge*), the local current density becomes excessive, leading to an electro‐chemo‐mechanical breakdown at the SSE/Na interface, resulting in dendrite intrusion through the dense ceramic [7, 8]. Therefore, improvements to anode stability and achievable current density are crucial to enable practical alkali‐metal SSBs such as sodium solid‐state batteries (Na‐SSBs).

**SCHEME 1:**
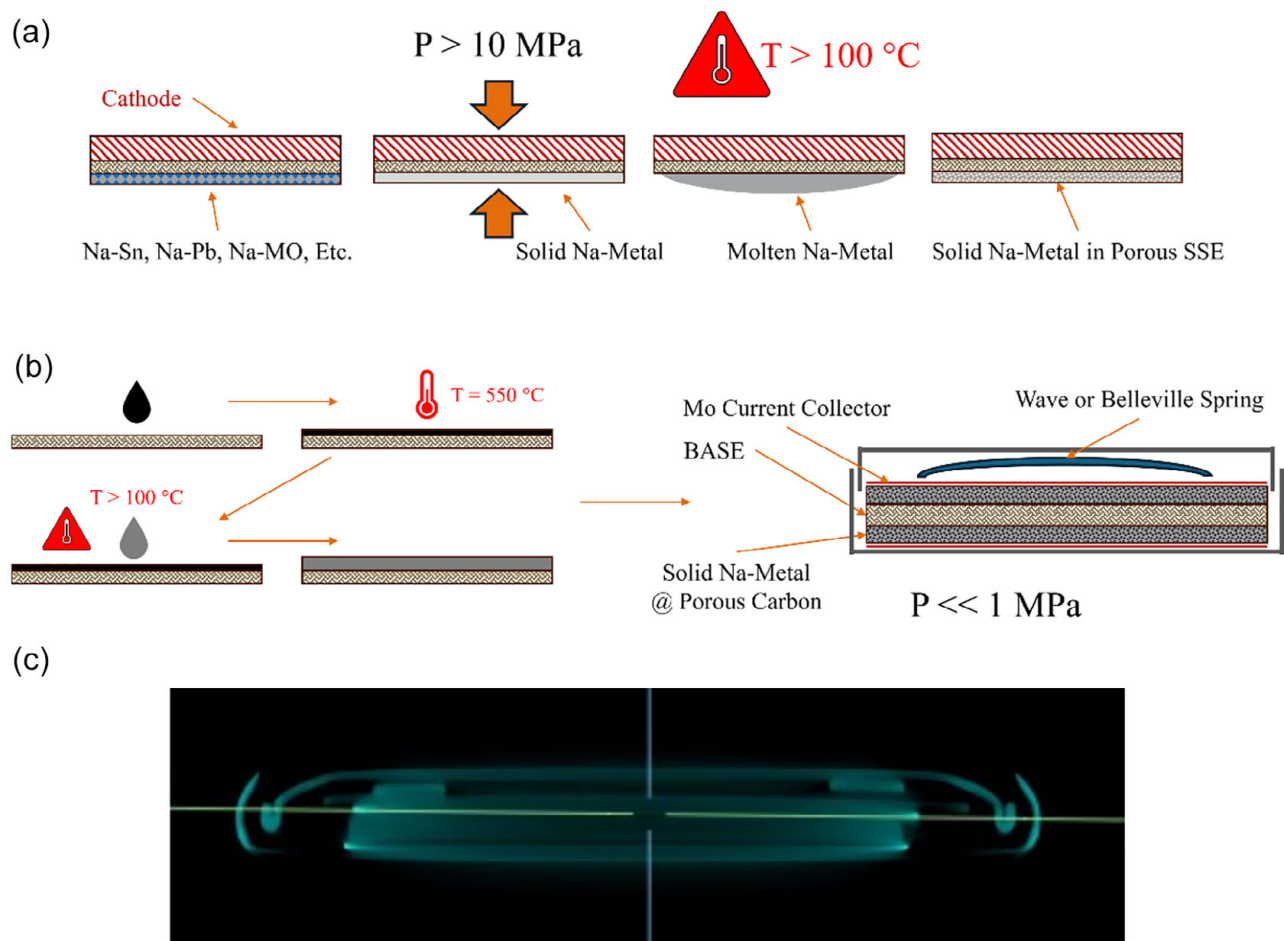
Strategies for robust Na‐metal anodes (a) commonly described in the literature, (b) presented in this work and tested in a reliable coin cell format, and (c) 2D image of Na–Na symmetric cell obtained from X‐ray microscope showing the cell architecture used in this study.

Many strategies have been employed in the literature to address the issue of poor contact between Na metal and the anode‐facing SSE (and resultant propensity to form dendrites). Generally, one of several distinct approaches to improve contact between Na and the SSE is employed including 1) formation of a sodiophilic interphase via alloying [9] or other spontaneous chemical reactions with an interfacial layer and Na‐metal [10, 11, 12, 13, 14, 15, 16, 17], 2) application of significant (often > 10 MPa [6, 7, 18, 19]) stack pressure during testing to leverage the ductility and fast creep‐induced [7] diffusion of Na metal to outpace the stripping current, and 3) utilize a molten alkali metal electrode with concomitant extremely fast liquid phase diffusion either at elevated temperatures (such as in molten Na‐S or ZEBRA batteries) [20, 21, 22, 23, 24, 25] or via eutectic alloys such as Na‐K [26, 27]. Variations on these approaches or combinations of them can be used to further improve anode cyclability such as was demonstrated by Jacshin et al. [16], where a sodiophilic ZnO interlayer along with a porous anode SSE layer was used to enable high geometric current densities with low local current density. Other emerging examples, such as the use of a Na‐ion‐conducting, Na‐metal‐stable polymer interphase, offer an alternative strategy to the aforementioned more common strategies to enable good contact between Na metal and an SSE [28]. Graphical depictions of the most common of these various approaches to enabling reliable Na‐metal anode behavior are shown in Scheme 1a. Finally, thermal treatment to produce an “ultraclean” surface on the SSE via removing contaminants such as carbonates or hydroxides, such as in the works of Bay et al. [5] for BASE or Sharafi et al. [4], as applied originally to Li‐conducting garnets, has been utilized to fundamentally understand the mechanism of poor interfacial contact.

Each of these aforementioned methods has merits and drawbacks. The use of spontaneous chemical reactions to form sodiophilic phases such as alloys or application of nanometric films of, for example, oxides [16] or sulfides [17] are effective, but generally utilize poorly scalable application methods (e.g., atomic layer deposition, ALD) or rely on relatively dense alloying [29] components (e.g., Sn [12, 15, 17, 25, 30], Pb [31, 32, 33], or Bi [34]), which will reduce the specific energy of the overall cell. The massive stack pressures utilized to enable reversible cycling and high areal capacity are obviously challenging for any practical battery pack due to the excess cost, complexity, and mass of the load frame necessary to apply such high pressure, where for state‐of‐the‐art batteries using, for example, prismatic rigid cell cases the initial stack pressure rarely exceeds several tens of PSI (i.e., <0.57 MPa) [35]. Molten Na electrodes are well known and effective, but the required elevated temperature places significant limitations on cell design and materials of construction, even for “intermediate” [36] temperature cell designs. Liquid alloy anodes such as Na‐K are extremely reactive, often being demonstrated to be incompatible with even highly robust oxide SSEs such as BASE or NaSICON [24], although some research indicates that Na‐K is stable [27, 37] under certain conditions such as lower operating temperature. Regardless, liquid alloy anodes also add a significant amount of inactive material in the form of the alloying component (generally K, though this is even more pronounced for, e.g., Na‐Cs [24] alloy anodes).

To this end, a different approach that leverages our previous [38] efforts to utilize a low‐cost, low‐mass porous carbon interfacial layer for molten Na batteries is explored in the context of a solid Na anode. While many of the aforementioned modifications have similarly utilized some form of carbon as an interfacial modification, they have either focused on molten Na batteries (e.g., Li et al. [33] and Landmann et al. [39]) or still suffer from a low CCD (e.g., Zhang et al.) [13]. The initial exploration of the so‐called Metal‐Free Wetting Layer (MFWL) previously showed excellent performance for molten Na even at temperatures just above the melting point of Na, driven by rapid and complete wetting of the porous carbon interlayer to produce a pristine interface between molten Na and the underlying BASE electrolyte [38]. Crucially, preliminary solid‐state Na cycling results showed that this MFWL could enable reasonable cycling in Na–Na symmetric cells, with a CCD of ∼1.8 mA cm^−2^. In this work, a variation of this porous‐carbon‐Na host matrix is demonstrated using the same application method, utilizing a simple coin‐cell symmetric cell design with mild stack pressures produced by either light or stiff springs (Scheme 1b). The cell architecture of an as‐assembled cell is revealed in Scheme 1c via X‐ray computed tomography (CT) microscopy. This cell design is used as a simple and repeatable test platform to understand the properties and limitations of this porous carbon anode host for SSBs, showing that cycling at reasonable current densities (>3 mA cm^−2^) and/or areal capacities (1 mAh cm^−2^) is achievable. The effects of testing temperature and pressure (via lighter or stiffer springs) are also explored. At a high level, the porous carbon anode host shows excellent resistance to dendrite formation, with a CCD greater than 10 mA cm^−2^ at 25 °C (0.25 mAh cm^−2^) and further reveals that even after significant cell polarization (due to void formation on stripping), dendrites can be largely prevented. Additionally, the results herein indicate that access to deeper cycling and higher areal capacity (>1 mAh cm^−2^) remains challenging in spite of its high CCD, motivating further improvement/optimization of the porous carbon layer to improve Na diffusion for Na‐SSB applications.

## 2 Results and Discussion

### 2.1 Initial Exploration

**FIGURE 1:**
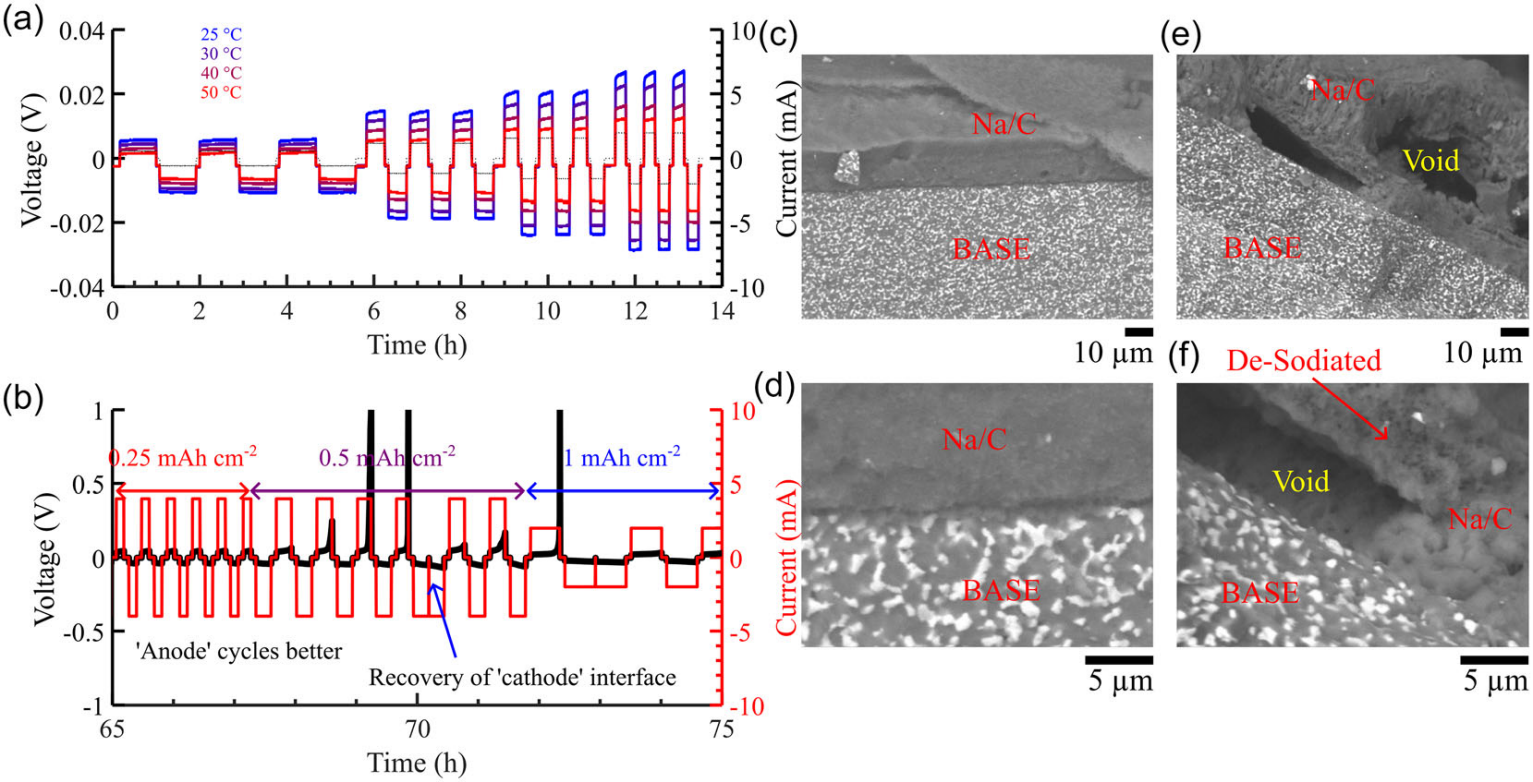
Exploratory symmetric cell testing using Version 1 porous carbon coating showing (a) current staircase experiment showing stable cycling up to 3 mA cm^−2^ (0.25 mAh cm^−2^) (b) cycling at various current densities and areal capacities to understand limits of cell performance at 50 °C, and low and high magnification backscatter electron (BSE) images of fracture surface of uncycled (c,d) and post‐cycled (e,f) Na–Na symmetric cells.

Initially, a modified ink formulation relative to our previous work was explored using ethanol (EtOH) as the only solvent to remove any effects due to water on the BASE SSE, since solid electrolytes like BASE_[5]_ and NaSICON [40] are known to be hygroscopic and will react with moisture and atmospheric CO_2_ to form sodiophobic [5] Na_2_CO_3_. This “Version 1.0” (described in the **Experimental Section**) formulation, like our previous MFWL coating, showed excellent Na wettability when molten Na was applied to the surface. For symmetric cells assembled as in Scheme 1, very low resistance was achieved (Figure S1) leveraging both the high ionic conductivity (>2 mS cm^−1^) of our BASE composite SSE and the exceptional interfacial contact between Na and BASE facilitated by the porous carbon host. The initial “current staircase” cycling (0.25 mAh cm^−2^ cycling capacity, Figure 1a) at various temperatures (20–50 °C) shows highly stable cycling up to 1 mA cm^−2^. In order to assess the behavior of this anode system while preventing failure due to dendrite intrusion, the same cell was cycled at various current densities and areal capacities at 50 °C (Figure 1b, with full voltage–current data plotted in Figure S2). The cell easily handled current densities up to 3 mA cm^−2^ at 0.25 mAh cm^−2^ and cycled reversibly for many cycles at 2 mA cm^−2^ with no obvious degradation. However, increasing the cycling capacity to 0.5 mAh cm^−2^ caused the cell to polarize past the +/−1V cutoff on “charge” before passing the targeting capacity. Despite this polarization, subsequent “discharge” did not produce a dendrite, rather showing an initial voltage polarization and subsequent decrease in voltage as more Na‐metal was plated onto the “cathode.” Indeed, doubling the discharge capacity to 1 mAh cm^−2^ allowed subsequent charge cycles to pass the full 0.5 mAh cm^−2^ capacity without reaching the voltage cutoff. On the other hand, reducing the current density to 1 mA cm^−2^ did not fully prevent the cathode from polarizing to 1 V. To test the limits of lower current density, the cell was heated slightly from 50 °C to 60 °C and cycled at 0.15 mA cm^−2^. Initially, the cell was able to cycle up to 3 mAh cm^−2^, but eventually the cell polarization grew, limiting the accessible capacity while still not resulting in a short circuit due to dendrite formation (Figure S3).

To understand the evolution of the Na electrodes after cycling, Cell 1 was disassembled, fractured, and characterized by scanning electron microscopy (SEM). Backscatter electron (BSE) images of the fracture surface of an uncycled Na electrode are shown in Figure 1c,d, while BSE images of Cell 1 after cycling are shown in Figure 1e,f. As expected, the uncycled cell shows perfect adhesion and surface contact between Na and the underlying BASE electrolyte. However, notable morphological changes after cycling are observed (Figure 1e,f) where some regions of maintained contact are noticeable, while significant void formation is also observed. In parallel, some regions of the porous carbon appear intact, albeit with more obvious pore structure visible, indicating significant physical desodiation. Interestingly, some of these voids can be observed deeper in the Na electrode, with fresh Na still present on the BASE surface. We hypothesize that, in areas where adhesion between BASE and Na (or more properly, the Na‐infiltrated porous carbon) is weaker, it is possible for the plating pressure of Na to push the electrode composite away from the BASE surface, thereby resulting in voids propagating into the Na/C electrode and away from the BASE surface. Simultaneously on the stripping side of the symmetric cell, areas where the porous carbon layer has lost adhesion are expected to more readily form these so‐called Kirkendall [41, 42, 43] voids. Similar effects were observed by Jolly et al. [7] and Wang et al. [41] in the case of pure Na electrodes and Li electrodes, respectively, depending on the applied pressure to the cell. In addition to the galvanostatic cycling data and SEM morphological characterization, electrochemical impedance spectroscopy (EIS) corroborates the interpretation of void formation and concomitant reduction in accessible surface area for Na plating/stripping, with a notable increase in overall cell impedance (Figure S4) observable. However, no dendrite‐induced short circuit was observed in the cell, even after repeatedly polarizing to significant voltages due to stripping current density outpacing the ability of fresh Na metal to replenish the Na/BASE interface. Notably, in Figure S4, the diffusion tail at low frequency is larger than before repeated plating and stripping, and an intermediate‐frequency charge transfer impedance is newly observed. We tentatively attribute these changes to the larger diffusion distance of Na within the Na‐metal electrode and the traversal of Na metal into and through the porous carbon, respectively, a conclusion corroborated by the observation of some porous structures visible in the Na/C electrode, both adhered to BASE and pushed away in Figure 1e,f. From these observations, we conclude that the presence of some still‐adhered porous (though “de‐sodiated”) carbon, which itself is an excellent electronic conductor, helps to distribute the current density on subsequent plating and prevent a localized current “hotspot” and resulting dendrite intrusion. This hypothesis is supported by the observation that, on reversal of current polarity, there is an initial “spike” in voltage (positive or negative depending on cycle conditions) followed by equilibration to a lower overpotential, indicating the replenishment of Na‐metal on the plating side of BASE to recover previously lost active surface area (Figure S5).

We briefly note that, despite the better dispersibility of carbon black in alcohols compared to aqueous solutions, the uniformity of the porous carbon layer resultant from the “Version 1.0” baseline still needed some improvement. As in our previous [38] work with an aqueous ink, a surfactant was added to improve carbon black dispersion and uniformity at the BASE interface, producing a “Version 2.0” ink as described in the **Experimental Section**, which is used in all other cells carbon treated hereafter. These 10 Na–Na symmetric cells were initially characterized with EIS at room temperature after assembly to confirm qualitative comparability, as plotted in Figure S6.

**FIGURE 2:**
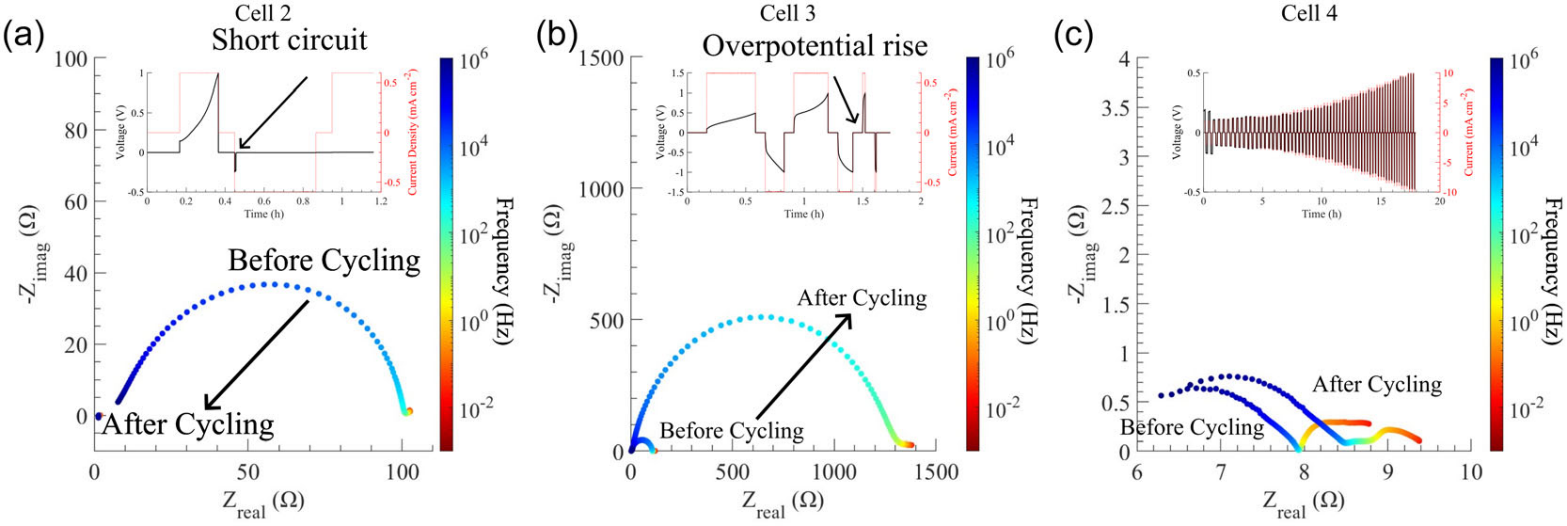
Comparison of EIS spectra of untreated BASE symmetric cells tested at (a) 25 °C and (b) 60 °C (voltage/current‐time traces shown in insets) versus (c) interface treated (“Version 2”) BASE electrolyte subjected to symmetric cycling showing EIS spectra before and after symmetric cycling test up to 10 mA cm^−2^ (inset).

The resistance of the porous carbon Na anode to dendrite formation is a significant benefit, especially compared to bare BASE symmetric cells assembled as a baseline. For example, using a fixed cycling capacity of 0.25 mAh cm^−2^, an uncoated BASE symmetric cell (application of Na metal described in the **Experimental Section**) failed due to an obvious short circuit at only 0.6 mA cm^−2^ on the first discharge at 25 °C (Figure 2a). A zoomed EIS spectrum of the cell post‐dendrite failure is shown in Figure S7. Interestingly, when cycling a symmetric cell assembled without a porous carbon host layer at 60 °C rather than 25 °C, failure due to dendrites was not observed—instead, high cell polarization functionally caused the cell to stop being able to plate and strip Na metal (Figure 2b) with a concomitant significant rise in cell impedance as measured by EIS. Alkali metals are well known to undergo significant creep at room temperature due to their low melting points [7, 41] (homologous temperature for Na‐metal T_H_ ∼ 0.8, for comparison T_H_ ∼ 0.66 for Li‐metal), where T_H_ is well above 0.4. We suspect that at 60 °C (T_H_ ∼ 0.9), the creep rate may be sufficiently high to enable rapid distribution of freshly plated Na metal to prevent dendrite initiation, even though apparently it is still too low to enable stripping without rapid void formation. Most likely, the increased Na‐ion conductivity of BASE at 60 °C (σ_60_ ∼ 7 mS cm^−2^, Figure S1) also plays a beneficial role in ensuring that the high flux of Na^+^ to the interface matches the plating current density and facilitates more uniform plating. In contrast, a symmetric cell utilizing the porous carbon host (“Version 2.0”) anode was able to cycle at 25 °C up to 10 mA cm^−2^ without any failure due to short circuit (Figure 2c). Critically, the ability to handle 10 mA cm^−2^ puts this Na‐porous‐carbon anode system in the range of fast charging goals in terms of current density (5 mAh cm^−2^ at 2C) [1], albeit at a lower cycling capacity. Doubling the cycling capacity to 0.5 mAh cm^−2^ did not cause failure due to dendrite intrusion, but rather showed the same overpolarization behavior as seen in Figure 1b (cycling data for Cell 4 with 0.5 mAh cm^−2^ capacity shown in Figure S8). Despite this, no short circuit was observed even at 5.5 mA cm^−2^ (though the cell struggled to deliver the full charge capacity above ∼3.4 mA cm^−2^), in contrast to the bare Na‐metal cell, confirming the general trend that the porous carbon anode can handle significant current density and resists dendrites, but struggles with deep cycling. Notably, while the cell impedance rose slightly after cycling (in line with observations from Cell 1), the rise is relatively minor. However, a new small charge transfer impedance, not observed in as‐assembled cells, is observable at intermediate frequencies (∼1 kHz to 1 Hz), an effect also observed in Cell 1 (Figure S4). The origins of this will be discussed afterward.

### 2.2 Systematic Exploration of Cycling Parameters

**FIGURE 3:**
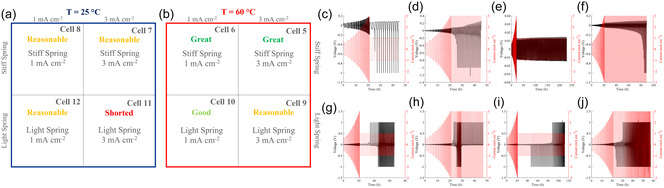
Testing conditions for exploration of effect of pressure and current density at fixed cycling capacity of 1 mAh cm^−2^ at cycling temperatures of (a) 25 °C and (b) 60 °C, and voltage/current versus time traces of (c) Cell 8, (d) Cell 7, (e) Cell 6, (f) Cell 5, (g) Cell 12, (h) Cell 11, (i) Cell 10, (j) Cell 9.

With initial results showing cycling stability at a high current density of 10 mA cm^−2^ juxtaposed with challenges with higher cycling capacity above 0.5 mAh cm^−2^, a systematic exploration of the effects of cycling current density, areal capacity, temperature, and pressure was performed. For each condition, a fresh, uncycled Na–Na symmetric cell was assembled. While some cell‐to‐cell variability is observable (see Figure S6, which shows EIS spectra of each cell as assembled at room temperature) based on differences in thickness of each BASE disc and completeness of surface coverage of the carbon layer, the cells used in this study are highly comparable. Briefly, temperatures of 25 and 60 °C (chosen based on results showing cell failure due to dendrites at 25 °C and lack of similar failure at 60 °C), low and high pressures (effectuated by light wave springs or stiffer Belleville springs, *P* < ∼0.72 MPa based on maximum crimping pressure and cell geometric area, see discussion in the Supporting Information), and maximum current density of either 1 or 3 mA cm^−2^ with a fixed cycling capacity of 1 mAh cm^−2^ were investigated to understand the conditions at which the cell is either unable to achieve full utilization or fails due to dendrite intrusion. These conditions are summarized in Figure 3a,b and Table S1. Discussion of criteria to assess the performance of the Na‐porous‐carbon anode under each testing condition is included in the Supporting Information. Each voltage/current‐time trace for all cells summarized in Figure 3a,b is plotted in Figure 3c–j, and all cells were initially subjected to the same “test” cycling protocol up to 3 mA cm^−2^ at a fixed cycling capacity of 0.25 mAh cm^−2^ before testing at the target condition in order to verify cell quality and comparability. Despite variable performance at higher cycling capacity, all cells easily passed the initial “current staircase” test with no issues.

The majority of cells cycled with no dendrite failure under these cycling conditions, with the exception of Cell 11 (low pressure, 25 °C, 3 mA cm^−2^). In contrast, other cells had varying degrees of success in utilizing the full 1 mAh cm^−2^ capacity under their respective testing conditions. Unsurprisingly, Cell 6 (higher pressure, 60 °C, 1 mA cm^−2^, Figure 3e) performed the best, showing stable cycling of 1 mAh cm^−2^ capacity for over 95 cycles before it was stopped for subsequent characterization. This accords with expectations as higher pressure facilitates Na transport largely via creep [7], and noting that creep rate is strongly temperature‐dependent. Evidently, under these conditions, the current density of 1 mA cm^−2^ is lower than the maximum intrinsic Na diffusion rate [6, 7], showing one strategy to enable a reversible solid‐Na anode. We do note, however, that some polarization is observable toward the end of each 1 mAh cm^−2^ cycle, though this does not appear to result in irreversibility on subsequent cycles. Cell 10 (Figure 3i), a replicate of Cell 6 with a lighter spring, also showed reasonable reversibility initially, though the cell polarization increased with increasing cycle number, indicating that too little pressure limits the long‐term reversibility of the cell even with the sodiophilic carbon treatment and elevated temperature. On the other hand, a lower cycling temperature of 25 °C and a stiffer spring showed better performance compared to a lighter spring and elevated temperature (Cells 7 and 8, Figure 3c,d). A more detailed comparison of the cells shown above is provided in Table S2. While pressure and temperature are clearly synergistic in improving the reversibility of the anode, pressure appears to confer relatively greater benefits. For this reason, the low‐pressure, elevated‐temperature strategy can be considered a beneficial strategy but is not a perfect solution to enable a reversible solid Na anode, especially for cells with higher areal capacities.

**FIGURE 4:**
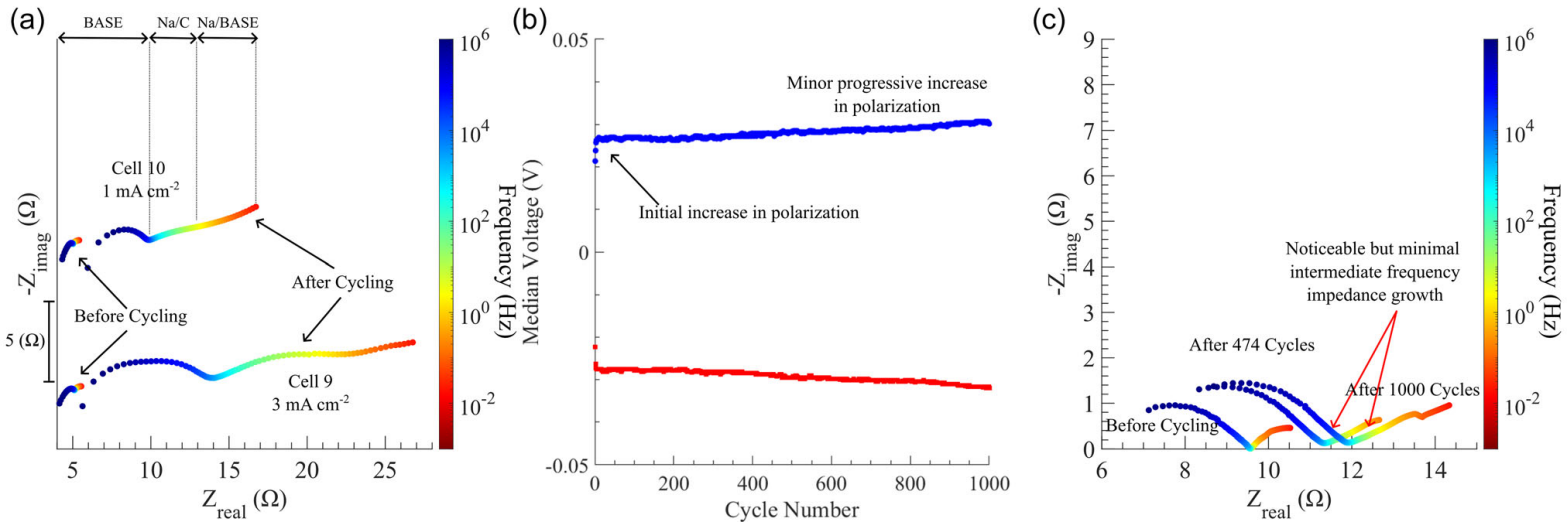
(a) EIS spectra of Cells 9 and 10 before and after galvanostatic cycling, revealing a greater overall increase in impedance for Cell 9 (higher current density), (b) median voltage for Cell 13 cycled at 25 °C, lighter spring, 1 mA cm^−2^, 0.25 mAh cm^−2^ showing initial increase in polarization followed by minor increase over 1000 cycles, and (c) EIS spectra of Cell 13 before cycling, after 474 cycles, and at the end of 1000 cycles.

Cells 9 and 10 (1 or 3 mA cm^−2^, 60 °C, lower spring pressure, Figure 3i,j) provide particularly useful information on the mechanism of overpotential rise with extended cycling and polarization, which, in some cases, can result in the inability to deliver the full 1 mAh cm^−2^ capacity, as the lighter pressure evidently accentuates the degradation behavior of the Na/C‐BASE interface. Cycling data unsurprisingly reveal that lower current density allows better utilization for more cycles before each electrode begins to polarize. However, EIS analysis confers better insight into the reason for electrode polarization: Figure 4a shows that, before cycling, Cells 9 and 10 have a nearly identical impedance response, with only a high frequency charge transfer impedance from the BASE SSE itself (based on the analysis of Bay et al.) [5], and a minimal low frequency charge transfer arising [5] from the Na‐BASE interface. However, after cycling, both cells show increased impedance including a similar intermediate (1 kHz–1 Hz) frequency charge transfer that is not present as‐assembled but is also observable in Cells 1 (Figure S4) and 4 (Figure 2c). The attributions of impedance processes from Na‐ion transport in BASE, Na‐metal transport in the porous carbon, and charge transfer at the Na/BASE interface are indicated in Figure 4a. Notably, Cell 9 (3 mA cm^−2^) shows a more significant increase in cell impedance relative to Cell 10. These various impedance responses derived from EIS analysis are listed before and after cell cycling in Table S3. Briefly, the BASE contribution to impedance for Cells 9 and 10 increases by ∼2.8x and 1.9x, respectively, after cycling, while the overall cell impedance increases by ∼4.9x and 3.1x, respectively. However, the intermediate frequency component that arises after cycling (not appreciably present as assembled) for Cell 9 is roughly 1.5x higher than for Cell 10. The void formation mechanism observed herein and in the literature offers a good explanation for both the increased high‐ and low‐frequency impedance responses for these cells, explained simply by a net reduction in actual electrochemically active surface area between the BASE SSE and Na metal. However, apparently the current density used for cycling affects the intermediate frequency charge transfer impedance more directly—indeed, this particular impedance is about 1.5x larger for Cell 9 than Cell 10, representing a more significant proportion of the overall impedance (∼20% for Cell 9 vs. ∼16% for Cell 10). Based on morphological characterization of Cell 1 (Figure 1e,f) showing desodiated porous carbon, and by the reliability of attribution of the high‐ and low‐frequency impedances to Na‐ion transport in BASE and the BASE/Na interface respectively, we hypothesize that the intermediate frequency charge transfer impedance represents the resistance to Na‐metal transport through the porous carbon towards/away from the BASE interface during Na‐stripping/deposition, respectively. This effect may arise either from sluggish/minimal Na‐intercalation into the porous carbon or from sluggish surface transport throughout the surface of the pore network. Further, this may explain the propensity in some cases for the carbon to be pushed away from the BASE interface during plating due to local pressure generated proportional to the plating current density, rather than traversing through the porous carbon and into the available void space. In short, higher current density appears to result in more rapid degradation of the interface between Na/C and BASE; this conclusion is not particularly surprising but does imply that improving the Na‐diffusivity within the carbon matrix is a crucial next step to enable higher current density operation at appreciable areal capacity utilization.

We note as well that most cells examined have one electrode that shows worse reversibility and a greater increase in polarization on continued cycling (e.g., Cell 10). We suspect that there is some minor difference in the heat treatment process based on which side of BASE is facing up versus down in the heat treatment furnace, that may affect, for example, the gas evolution or carbonization of the polymer/surfactant component of the ink solution during pyrolysis. Anecdotally, it seems that the side facing downward tends to exhibit slightly slower Na wetting, which we attribute to slower gas evolution/pyrolysis during the heat treatment. However, this drawback is not expected to affect full cells, where only one side is treated with the porous carbon interfacial treatment, as will be discussed subsequently. Although we can presume that the general behavior of the anode platform will comport more closely with the “better” cycling behavior (charge or discharge) shown in the voltage profiles in Figure 3, we include the “worse” cycling behavior in interpretation of the cell data summarized in Figure 3a,b, while noting that all cell testing conditions with “reasonable” performance or better are likely to succeed in a full cell configuration.

Next, the long‐term cyclability of the porous carbon Na host was tested at 0.25 mAh cm^−2^ cycling capacity and 1 mA cm^−2^ current density (Figure 4b). In this case, Cell 13 (lighter spring, 25 °C) was able to cycle for 1000 cycles with an increase in overpotential of ∼48%. EIS spectral analysis (Figure 4c) shows the general slow degradation of this cell, resulting in an increase in high‐frequency impedance from ∼9.5 Ω to ∼11.5 Ω after 474 cycles and to ∼12 Ω after 1000 cycles. However, it is notable that this increased cell resistance (as measured by the cell overpotential) stabilized after only five cycles, followed by a more incremental increase over all cycles. Based on the mechanism of void formation and incomplete filling on multiple plating and stripping cycles, this is most readily attributable to an initial irreversible loss of a fraction of the contact area between Na and BASE, stabilization of the interface after this initial void formation, and finally a gradual but minor loss in contact area between the Na‐porous‐carbon anode and BASE.

### 2.3 Structural Evolution of the Porous‐Carbon‐Sodium Anode After Cycling

**FIGURE 5:**
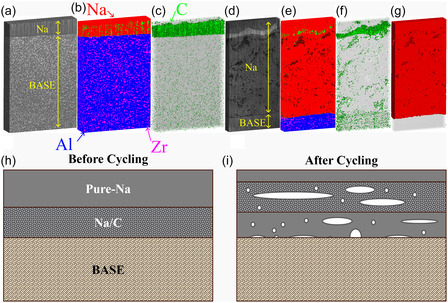
Comparison of Na/C/BASE cells showing pristine sample (a) BSE tomogram revealing BASE and Na regions, (b) EDS map reconstruction of probed volume in (a) showing Na‐ß″‐Al_2_O_3_/YSZ composite, Na/C composite, and perfect interfacial contact between the two, (c) C‐K X‐ray signal reconstruction showing morphology of porous carbon interfacial layer (note that C‐K X‐ray signal in BASE region is likely due to some redeposition during cryo‐PFIB sectioning), and cycled sample (d) BSE tomogram revealing BASE and Na regions with significant morphological evolution of the Na/C electrode, (e) EDS map reconstruction of probed volume in (d) highlighting the formation/propagation of voids into the Na/C electrode and, most notably, the traversal of the porous carbon layer away from the BASE interface, (f) C‐K X‐ray signal reconstruction confirming the traversal of the majority of the porous carbon away from BASE, with some carbon retained in the pore walls of void spaces/minimally at the BASE interface, (g) Na‐K X‐ray signal highlighting the morphology and distribution of voids in the electrode after extended cycling, and finally graphical depictions of proposed structural evolution mechanism showing (h) pristine and (i) cycled structures (Red—Na, Blue—Al, Green—C, Magenta—Zr X‐ray signals).

To assess the structural evolution of the BASE/Na/C interface, cryogenic plasma focused‐ion‐beam (cryo‐PFIB) cross‐sectioning and tomography with energy dispersive X‐ray spectroscopic (EDS) mapping were performed (Al—blue, Na—red, C—green, and Zr—magenta used for elemental mapping). As a baseline, a sodiated but uncycled cell was analyzed with cryo‐PFIB cross‐sectioning, with representative tomogram and EDS map reconstructions shown in Figure 5a–c. As expected, the contact between the Na/C‐layer and underlying BASE SSE is essentially perfect in the tomogram (Figure 5a) and reconstructions (Figure 5b,c), an observation confirmed by examination of each sequential “slice” from ion milling and SEM‐EDS shown in Movie M1. (Note that the C‐K‐ray signal was weighted in the data analysis to allow it to be prominent in the tomographic reconstruction due to its low mass fraction and low atomic number). Notably, the C‐layer is completely filled with Na, and some excess Na is observed atop the C‐layer in contact with the current collector. For comparison, Cell 13 was disassembled and examined in the same way, with tomogram and EDS map reconstructions shown in Figure 5d–g. (Note that for Figure 5d–g, the region of interest focuses primarily on the Na anode, and only a small region of BASE is visible near the bottom of the reconstructions, opposite Figure 5a–d).

Clearly, significant evolution of the Na‐electrode occurred during cycling, with many voids apparently being pushed away from the surface of the BASE electrolyte (Figure 5d). Strikingly, in the composite EDS reconstruction (Figure 5e), the C‐layer is observed to have traveled away from the Na/BASE interface, ∼50 µm into the Na layer. Some C‐K X‐ray signal is observed in the pore walls in the voids in the Na layer (Figure 5f), and regions of maintained contact and local voids are observable at the interface between the Na layer and BASE, not to mention the aforementioned voids that have formed in the bulk of the Na‐metal anode (Figure 5g). Based on the movement of the porous C‐layer away from the BASE, we postulate that at these mild testing pressures, repeated plating and stripping of Na metal results in a migration of both the interfacial porous C‐layer and the Na metal away from the BASE. This morphology is relatively homogeneous, as observed in the slice‐by‐slice movie of the reconstructed tomogram from Cell 13 (Movie M2) and crucially still shows a reasonably large contact area between the Na layer and BASE SSE.

Despite the overall deleterious formation of voids under lower pressure and at 25 °C, Cell 13 did not fail due to dendrite‐induced short circuit over 1000 cycles, indicating that the porous carbon interface and overall anode arrangement prevent the kind of rapid short circuit observed in Cell 2 under similar conditions. Currently, we believe that, corroborated by SEM observations of Cell 1 and PFIB‐tomography of Cell 13, some of the porous carbon helps to maintain contact at the interface, thereby allowing current density to be distributed on plating and preventing locally excessive current density and resultant dendrite initiation. In parallel, the initial near‐perfect contact between Na and BASE afforded by the sodiophilic porous carbon layer appears to maintain some cohesion of the electrode after void formation, even in regions where the porous carbon delaminates and traverses away from the interface. This, combined with minimal but nonzero pressure from the wave spring and the ductility of Na metal, appears to allow reasonable electrode reversibility over 1000 cycles with only minimal increase in resistance and no dendrite‐induced short circuit.

### 2.4 Full Cell Using Na/BASE Anode/Separator and Layered Oxide Cathode

**FIGURE 6:**
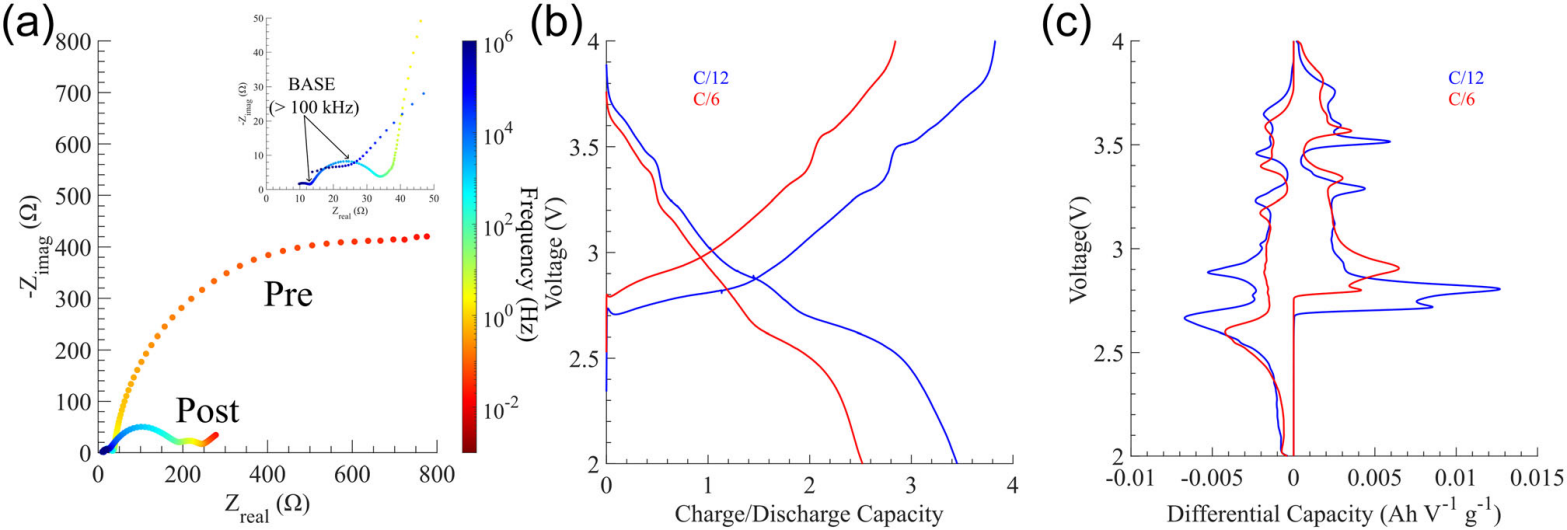
Full cell data for Cell 14 with Na/C anode and BASE separator and Na‐TMO cathode showing (a) EIS spectra before and after cycling Cell 14 showing reduction in significant low frequency impedance after initial cycling/conditioning of the cell and showing expected low contribution of the Na/C/BASE anode to overall cell impedance (inset), (b) voltage‐capacity profiles under galvanostatic cycling conditions at C/12 (0.125 mA cm^−2^) and C/6 (0.25 mA cm^−2^) (based on capacity of cathode) showing discharge capacities of 2.71 and 1.97 mAh cm^−2^ respectively, and (c) related differential capacity plot showing expected phase transitions of the Na_0.92_Mn_0.5_Ni_0.4_Fe_0.1_O_2_ cathode.

In order to test the ability of the Na/C anode system in a full cell configuration, a quasi‐solid‐state cell was assembled using a sodium transition‐metal‐oxide (Na‐TMO) cathode and minimal (30 µL) liquid electrolyte on the cathode side. Specifically, Cell 14 was assembled, utilizing a high areal capacity Na_0.92_Mn_0.5_Ni_0.4_Fe_0.1_O_2_ layered oxide cathode [44] (∼3 mAh cm^−2^, ∼3.8 mAh total capacity), tested at 25 °C, exhibits the expected low impedance response at high frequency, corresponding to the Na/BASE anode, with a higher impedance at low frequencies (Figure 6a). This indicates that the anode contributes very little to the cell impedance, similar to symmetric cells. We believe that the intermediate frequency impedance response in this case most likely arises from the formation of a resistive interphase between BASE and a localized high concentration electrolyte (LHCE) used, comprising sodium bis(fluorosulfonylimide) (NaFSI), dimethoxyethane (DME), and bis(2,2,2‐trifluoroethyl)ether (BTFE) as the cathode‐facing supporting electrolyte [44]. Meanwhile, we suspect that the relatively higher low‐frequency impedance results from initial imperfect wetting of the LHCE into the high‐capacity, calendared cathode layer. Indeed, after cycling, this low‐frequency impedance reduced significantly (Figure 6a).

Regardless, the cell was able to cycle at low current density (0.125 mA cm^−2^, ∼C/12) and achieved ∼100% utilization on the first charge (Figure 6b), while discharging 90% of the total capacity (∼3.44 mAh out of 3.82 mAh total). Compared to the challenges the Na‐carbon‐BASE anode exhibited at higher current density, lower current density allows very high utilization of the Na inventory in the anode (2.71 mAh cm^−2^) based on the lower area of the cathode relative to the anode. At 0.25 mA cm^−2^, the cell was still able to discharge 1.97 mAh cm^−2^ (Figure 6b). Finally, the expected phase transitions [44] of the Na‐TMO cathode are clearly observable both in the voltage‐capacity plots as well as differential capacity (“dQ/dV”) plots in Figure 6c. While anode polarization likely plays some role, the cathode is most clearly limited in terms of feasible current density based on the much greater impedance arising from processes unrelated to ion conduction in the BASE SSE or interfacial charge transfer impedance from the Na/C/BASE interface (Figure 6a). Though this cell is just a test case and not a true SSB, it highlights the reliability of the anode platform to handle larger areal capacity when paired with a suitable cathode system. Future efforts to develop all‐solid cathode composites are the subject of ongoing research.

## 3 Discussion

As shown above, both high current density (>10 mA cm^−2^) for short durations (i.e., 0.25 mAh cm^−2^) (Figure 2c) and moderate current density (1–3 mA cm^−2^) with capacity up to 1 mAh cm^−2^ (Figure 3e,f) are achievable at mild applied pressures (<0.72 MPa). Nonetheless, achieving the combination of high current density *and* high areal capacity—an ideal scenario for practical Na‐SSBs—appears to be unattainable with this current iteration of the Na‐porous‐carbon anode, requiring further innovation in materials and cell design. Despite this, cell failure due to dendrite initiation is *largely prevented,* and the primary failure mode of cells is cell polarization, which prevents full capacity utilization. This failure mode, while requiring further innovation to ameliorate, is still highly preferable to a short circuit.

In the broader context of current scientific literature on Na‐metal anodes, the results presented herein compare well, with a CCD comparable with many of the better Na‐metal anode embodiments outlined in Table 1. In terms of reversible areal capacity, our Na‐porous‐carbon anode also compares relatively well, especially given the low operating pressure. By comparison, most of the methods achieving higher areal capacity rely on a significant applied pressure during cell testing (e.g., Bay et al. – 3.4 MPa [5], Jolly et al. – 9 MPa [7], Ma et al. – 6–12 MPa [46], Deyscher et al. –10 MPa [18],). Tsai et al., on the other hand, operated a NaSICON‐based symmetric cell with no applied pressure during cycling, achieving an impressive CCD of 12 mA cm^−2^ and a high areal capacity of 5 mAh cm^−2^ at 1 mA cm^−2^ [6]. However, we note that initially, a large stack pressure (12 MPa) was applied to bond Na metal to the NaSICON substrate, which conferred near‐perfect contact at the interface.

**TABLE 1:** Comparison of cycling parameters on critical current density, accessible areal capacity, and cycle life on Na‐anodes from various sources in the Na‐SSB literature.

Cell type	Temperature, C	Pressure, MPa	CCD/Capacity, mA cm^−2^/mAh cm^−2^	Areal Capacity/Current density, mAh cm^−2^/mA cm^−2^	Cycle life	Reference
Symmetric–Na‐ß″‐Al_2_O_3_	25 °C	3.4	12/0.25 5.5/3	—		Bay et al. [5]
Symmetric–Na‐ß″‐Al_2_O_3_	25 °C	9	2.5/0.75	—		Jolly et al. [7]
Symmetric–NaSICON	25 °C	∼0/12^a^	9/∼0.6	5/1	∼55	Tsai et al. [6]
Symmetric–NaSICON–Na/Sn alloy	RT	∼0	2.5/0.25	0.25/0.5	500	Oh et al. [12]
Symmetric–Na‐ß″‐Al_2_O_3_/YSZ composite	80 °C	∼0.18	∼7/∼3.5	—	—	Deng et al. [45]
Symmetric–NaSICON	25 °C	6–12	14/1.2	1/1	500	Ma et al. [46]
Symmetric–NaSICON	25 °C	∼0	0.6/0.6	0.5/0.5	250	Zhang et al. [13]
Symmetric–Na‐ß″‐Al_2_O_3_/YSZ composite	80 °C	∼0	0.8/−	0.3/0.3	100	Lu et al. [11]
Symmetric–NaSICON	30 °C		1.8/∼0.6^b^	0.1/0.3	2700	Yang et al. [28]
Symmetric–Na‐ß″‐Al_2_O_3_/YSZ composite	30 °C	<0.72	1.88/∼0.262	—	—	Weller et al. [38]
Symmetric–Na_4_ B_10_H_10_B_12_H_12_	RT	10	6/4	—	—	Deyscher et al. [18]
Full–Na/Na_4_ B_10_H_10_B_12_H_12_/ Na_0.625_Y_0.25_Zr_0.75_Cl_4.375_/ NaCrO_2_	40 °C	10	—	7/1	400	Deyscher et al. [18]
Symmetric–Na_3_Hf_2_Si_2_PO_12_–Na/K alloy	RT	NR	2.6/NR	—	—	Suo et al. [47]
Symmetric–NaSICON	60 °C	NR	2.4/2.4			Liu et al. [17]
Symmetric–NaSICON–sputtered Sn	60 °C	NR	3.8/3.8			Liu et al. [17]
Symmetric–NaSICON–sputtered SnS_2_	60 °C	NR	—	3/3	150	Liu et al. [17]
Symmetric–NaSICON–sputtered SnS_2_	RT	NR	—	2/2	375	Liu et al. [17]
Symmetric–trilayer NaSICON–ALD ZnO	RT	∼0	42/42	30/30	100	Jaschin et al. [16]
Symmetric–BASE	25 °C	<0.72	>10/0.25 >5.5/0.5	—	—	This work
Symmetric–BASE	25 °C	<0.72	—	1/0.25	1000	This work
Symmetric–BASE	60 °C	<0.72	—	1/1	>95	This work
Full–BASE/LHCE/Na‐TMO	25 °C	<0.72	—	0.125/2.72 0.25/1.97		This work

Abbreviations: NR, Not Reported; RT, Room Temperature.

a Cell assembled using 12 MPa pressure.

b Estimated from the figure.

In contrast, several works utilize little to no stack pressure, often relying on an alloying‐type anode to facilitate high current density or areal capacity. For example, Oh et al. [12] showed that a composite anode consisting of Na and Na_15_Sn_4_ has a CCD of 2.5 mA cm^−2^ (0.25 mAh cm^−2^ cycling capacity), and can cycle stably at 0.5 mA cm^−2^ for 500 cycles, owing in large part to fast Na‐diffusion in the Na_15_Sn_4_ intermetallic and the significantly improved wettability of the latter on NaSICON compared to Na alone. Yang et al. [28] also relied on the formation of a Na‐Sn alloy by utilizing a deposited SnF_2_ coating on NaSICON, which converted to NaF and Na‐Sn alloy upon exposure to molten Na, conferring a CCD of 1.8 mA cm^−2^ (9x increase from bare NaSICON) at 30 °C, and over 2000 cycles at a cycling capacity of 0.1 mA cm^−2^. Liu et al. [17] similarly utilized sputtered Sn or SnS_2_ on NaSICON as an anode interfacial layer, demonstrating a CCD of 3.8 mA cm^−2^, and more interestingly a deeper cycling capability of 3 mAh cm^−2^ (3 mA cm^−2^) at 60 °C and 2 mAh cm^−2^ (2 mA cm^−2^) at room temperature. Suo et al. [47] utilized a different approach of a liquid alloy anode comprising eutectic Na‐K and a K‐metal stable Hf‐containing NaSICON SSE, with a CCD of 2.6 mA cm^−2^ at room temperature. A different approach was utilized by Yang et al. [28], relying on an in situ electroinitiated polymerization to form a thin polymer electrolyte compatible with both Na metal and NaSICON. In this case, excellent coverage of the NaSICON interface facilitated intimate contact with Na metal and uniform current density, enabling a CCD of 6.8 mA cm^−2^ without applied pressure. Further, this strategy enabled 1 Ah pouch‐cell‐scale NaSSBs without applied pressure. Finally, advanced ceramic engineering utilizing a porous‐dense‐porous structured NaSICON membrane was demonstrated by Jaschin et al. [16] wherein an exceptional CCD of 42 mA cm^−2^ was achieved at room temperature with no applied pressure. Enabled by a well‐defined ZnO interfacial layer (effectuated by atomic layer deposition, ALD) in a porous NaSICON Na‐host layer, an actual Na‐NaSICON contact area significantly larger than the cell geometric area was achieved, which also beneficially reduced the diffusion distance between Na‐metal and the ion‐conducting NaSICON.

While many different approaches have shown a high CCD, a high areal capacity, or in some cases both, most rely on complex interfacial coating methods (sputter deposition, ALD, electrospray, etc.) or a significant proportion of an alloying component, which reduces the active material of the anode. One of the major goals of this work is to demonstrate an extremely simple coating process, relying on nontoxic, air‐stable, and low‐cost inks with only cheap carbon black as the sodiophilic component. In this work, the benefits of high CCD, dendrite resistance, and ease of application have been demonstrated, while also demonstrating excellent reversibility for intermediate areal capacities. Clearly, the resistance to dendrites at higher current densities (>10 mA cm^−2^) is not the major limitation of the Na‐porous‐carbon anode demonstrated herein; rather, limitations on deeper cycling are observed, although at low current densities, a quasi‐full SSB was still able to access ∼2.7 mAh cm^−2^. On a separate but crucial note, the adhesion of the porous carbon Na‐host on SSEs is fairly good but clearly degrades over many cycles. We have recently demonstrated a salt‐additive method for this porous carbon coating, which improves surface adhesion over the standard porous carbon coating, demonstrating one future pathway to reducing the propensity for freshly plated Na metal to push the carbon layer away from BASE [48]. The implication of traversal of the porous carbon layer away from BASE with extended cycling indicates that it primarily enables a near‐perfect initial interface between Na and BASE, which can degrade but still remains relatively cohesive after extended cycling and the pushing away of the carbon layer. With these results in view, methods to fine‐tune the porous carbon layer to improve adhesion as well as intrinsic Na‐metal transport are in progress to further extend the utility of this low‐cost Na‐host material for Na‐SSB operation at low stack pressures.

## 4 Conclusion

In this work, a new approach to the preparation of Na‐metal anodes for Na‐SSBs is described, based on hosted Na metal in a uniform and porous carbon matrix. Utilizing this easily applied porous carbon interlayer, symmetric cells utilizing a BASE composite solid electrolyte are able to handle a current density greater than 10 mA cm^−2^ at room temperature without dendrites, compared to only 0.6 mA cm^−2^ for uncoated BASE. Additionally, the relationship between testing temperature and pressure (using light or stiff springs as a proxy for low and high pressure) is explored, revealing that while both higher pressure and temperature can improve cyclability, pressure has a greater effect than increased temperature. Cells cycled at 60 °C and moderate pressure (<0.72 MPa) can reversibly cycle 1 mAh cm^−2^ at 1 mA cm^−2^ current density with minimal polarization, showing a strategy for mild‐pressure Na‐SSB design, which is crucial for practical SSB pack design. Further, the Na‐porous‐carbon anode facilitates stable long‐term cycling for 1000 cycles at low stack pressure at 25 °C. Interestingly, the repeated plating and stripping process over 1000 cycles results in propagation of much of the porous carbon away from the Na/BASE interface, along with formation of voids, which traverse away from the same interface. Despite this, sufficient interfacial integrity is maintained such that no dendrites form due to current focusing at the interface between Na and BASE. We propose that the high electronic conductivity of porous carbon facilitates the distribution of current density such that local current densities do not exceed a critical threshold, preventing dendrite‐induced short circuit. In parallel, a quasi‐solid‐state test cell using our Na‐metal anode system with a BASE separator was paired with a high capacity Na‐TMO cathode, showing utilization of ∼2.7 mAh cm^−2^ and ∼2 mAh cm^−2^ at C/12 and C/6 cycling rates respectively, delivering 90% of the theoretical capacity of the cathode at the C/12 discharge rate (after charging the full capacity on first charge), and demonstrating that the Na‐porous‐carbon anode system can operate at relatively high areal capacity loadings in a test cell operated at low stack pressure. The results presented herein serve as a launching point for low‐cost Na‐SSBs operated at low stack pressure for long‐duration stationary energy storage.

## 5 Experimental Section

### 5.1 Materials Preparation

Preparation of beta‐alumina solid‐electrolyte (BASE) disks used a modification of our previous [49] process wherein smaller BASE disks (∼2 cm^2^ area, ∼16 mm diameter, ∼0.5 mm thickness) than our standard electrolyte were prepared using the same vapor‐phase conversion process to form a 70 vol% Na‐β″‐Al_2_O_3_, 30 vol% Yttria‐stabilized Zirconia composite. The electrochemical properties of these ∼2 cm^2^ BASE SSEs are outlined in Figure S1.

### 5.2 Preparation and Heat Treatment of Porous Carbon Wetting‐Layers on BASE Electrolytes

The porous carbon wetting layers used in this study are based largely on our previous “metal‐free wetting layer” [38] with slight modifications. Briefly, carbon black (Vulcan XC‐72, FuelCellStore), poly(vinylpyrrolidone) (PVP, Sigma–Aldrich), and either glycerol (Sigma–Aldrich) for “Version 1.0” inks or Tergitol 15‐S‐9 (Sigma–Aldrich) for “Version 2.0” inks were dissolved/dispersed in pure ethanol (Koptec 200 proof anhydrous). After stirring overnight, the resultant ink was deposited via drop‐casting onto BASE electrolyte disks using 30 µL of ink on each side of the BASE disk for symmetric cells, and only on the anode side for a full cell. We note that this process results in some minor variation in actual surface area and that the more conservative nominal area of 2 cm^2^ is used throughout this study.

Subsequently, heat treatment was performed in flowing N_2_ in a CM Inc. Rapid Temperature Furnace. In order to enable both top and bottom sides of the substrate to have a uniform heating environment, the coated BASE disks were placed on glass substrates to raise them above the bottom of the furnace and provide a gap to enable gas evolution and transport during heat treatment. The heating schedule comprised a ramp to 120 °C at 3 °C min^−1^, followed by a hold for 1 h. Then, the substrates were heated to 550 °C at 3 °C min^−1^ for 5 h. Finally, the samples were cooled at 2 °C min^−1^ to 75 °C before being transferred into an Ar‐filled glovebox for cell assembly.

### 5.3 Assembly of Solid‐State Cells

Porous carbon‐treated BASE disks were placed on a hotplate in an Ar‐filled glovebox and slowly heated to 150 °C. Concurrently, several grams of Na metal were heated in a glass vial to the same temperature. Once the temperature had equilibrated, a glass pipette was used to transfer a small Na droplet from the glass vial Na reservoir onto a Mo disk. Then, the preheated BASE disk was placed onto the Na‐droplet‐on‐Mo assembly, resulting in immediate infiltration of the Na droplet into the porous carbon. This process also results in the Mo disc being pulled to the surface of the BASE disc due to capillarity. For Na–Na symmetric cells, another small droplet of Na metal was placed atop the other side of the BASE disc, which facilitated infiltration into the other porous carbon layer, followed by placing another Mo disk atop the entire assembly and slow cooling to room temperature. For quasi‐solid‐state full cells, instead of sodiating both sides of the BASE disk, only one side was sodiated before cooling. Then, a small amount (30 µL) of liquid electrolyte (localized high concentration electrolyte, LHCE, prepared as in Xiao et al. [44]) was pipetted onto a high‐loading (3 mAh cm^−2^, 1.27 cm^2^ area, total capacity of ∼3.8 mAh) electrode consisting of a layered‐oxide‐type Na_0.92_Mn_0.5_Ni_0.4_Fe_0.1_O_2_ cathode [44]. For untreated baseline cells, fresh Na‐metal chips (MTI International) were initially placed on top and bottom of a pristine BASE disc on a hotplate in an Ar‐filled glovebox. Then, a weight was placed on top of this assembly, and the hotplate was heated to 90 °C to enable the Na metal to soften and compress against the BASE surface. After 1 h of bonding, the Na‐BASE‐Na assembly was flipped so that the opposing electrode was also sufficiently bonded to the BASE surface. Cells assembled in this way had strong adhesion of Na metal to the BASE surface.

### 5.4 Materials Characterization

SEM imaging was performed on a JEOL IT‐200 SEM equipped with a JEOL energy‐dispersive X‐ray spectroscopy (EDS) detector or on a Thermo Scientific Helios Hydra UX dual‐beam plasma‐focused‐ion‐beam (PFIB) instrument equipped with an Oxford Instruments EDS detector. Generally, electron imaging was performed at 10 kV / 2 kV accelerating voltage on the IT‐200/Helios, respectively. Higher accelerating voltages were also used for the acquisition of EDS maps.

PFIB cross‐sectioning was performed using an Xe‐ion beam. Optionally, deposition of either Pt or W was performed with Xe ions at 12 kV accelerating voltage, and ion milling was performed at 30 kV. For sodiated samples, cryo‐PFIB milling was performed using a cryo‐stage, which was chilled using near‐liquid N_2_ temperature gas as the cryogen, to facilitate reliable milling of samples with Na metal. PFIB EDS tomography experiments were conducted.

Inert air transfer of sodiated samples to either the IT‐200 or the Helios was facilitated using sample holders designed to be sealed inside the glovebox and opened into an evacuated microscope chamber to prevent interaction of the sample with air.

A laboratory‐based X‐ray microscope (Zeiss Versa 610) was utilized to capture two‐dimensional (2D) images of the coin cell. The 2D imaging was conducted at a magnification of 0.4x, with the X‐ray source operating at an energy of 80 kV and a power of 10 W, using absorption‐contrast mode.

#### 5.4.1 Cell Assembly

Symmetric or full cells were assembled into standard CR2032 coin cells with relevant springs (light‐wave springs or stiff Belleville springs) and pneumatically crimped with a gas pressure of 65 PSI (∼0.45 MPa). This process was conducted in an Ar‐filled glovebox. Based on the ratio between the crimping piston and the BASE electrolyte disc, the maximum pressure on the electrode/BASE assembly applied during crimping is estimated to be ∼0.72 MPa (see discussion in the Supporting Information). This value serves as an upper limit on the internal pressure during cell testing, which is further modulated by the stiffness of the spring used. Once crimped, cells were removed from the glovebox for electrochemical testing.

#### 5.4.2 Electrochemical Characterization

Galvanostatic cycling tests were performed using a Landt battery cycler (CT3001A). Cells were tested at temperatures ranging from 10 to 60 °C using an isothermal chamber (TestEquity or Neware test chambers) to maintain temperature uniformity between +/‐ 0.1 °C. Potentiostatic EIS measurements were performed with a Gamry Reference 600+ potentiostat using a frequency range generally from 1 MHz to 0.02 Hz, or an Admiral Instruments Squidstat between 2 MHz and 0.02 Hz. An AC voltage amplitude of 10 mV was employed in all cases. Before each measurement, the cell was rested in order to reach thermal equilibrium.

#### 5.4.3 Data Analysis

Electrochemical impedance spectra were fit using ZView Software (Scribner), utilizing resistor (represented by “R”) and constant‐phase‐element (CPE, represented by “Q”) circuit elements.

## Funding

This work was supported by the Office of Electricity.

## Conflicts of Interest

The authors declare no conflicts of interest.

## Supporting information

The authors have cited additional references within the Supporting Information, which is accessible at (DOI).

Supplementary Material

Supplementary Material

## Data Availability

The data that support the findings of this study are available from the corresponding author upon reasonable request.

## References

[1] P. Albertus , S. Babinec , S. Litzelman , and A. Newman , “Status and Challenges in Enabling the Lithium Metal Electrode for High‐Energy and Low‐Cost Rechargeable Batteries,” Nature Energy 3 (2017): 16–21.

[2] D. Lu , Y. Shao , T. Lozano , et al., “Failure Mechanism for Fast‐Charged Lithium Metal Batteries with Liquid Electrolytes,” Advanced Energy Materials 5 (2014): 1400993.

[3] W. Xu , J. Wang , F. Ding , et al., “Lithium Metal Anodes for Rechargeable Batteries,” Energy & Environmental Science 7 (2014): 513–537.

[4] A. Sharafi , E. Kazyak , A. L. Davis , et al., “Surface Chemistry Mechanism of Ultra‐Low Interfacial Resistance in the Solid‐State Electrolyte Li_7_La_3_Zr_2_O_12_ ,” Chemistry of Materials 29 (2017): 7961–7968.

[5] M. C. Bay , M. Wang , R. Grissa , M. V. F. Heinz , J. Sakamoto , and C. Battaglia , “Sodium Plating from Na‐β″‐Alumina Ceramics at Room Temperature, Paving the Way for Fast‐Charging All‐Solid‐State Batteries,” Advanced Energy Materials 10 (2019): 1902899.

[6] C.‐L. Tsai , T. Lan , C. Dellen , et al., “Dendrite‐Tolerant All‐Solid‐State Sodium Batteries and an Important Mechanism of Metal Self‐Diffusion,” Journal of Power Sources 476 (2020): 228666.

[7] D. Spencer Jolly , Z. Ning , J. E. Darnbrough , et al., “Sodium/Na Beta” Alumina Interface: Effect of Pressure on Voids,” ACS Applied Materials & Interfaces 12 (2020): 678–685. 31815414 10.1021/acsami.9b17786

[8] L. Porz , T. Swamy , B. W. Sheldon , et al., “Mechanism of Lithium Metal Penetration through Inorganic Solid Electrolytes,” Advanced Energy Materials 7 (2017): 1701003.

[9] C. Wang , H. Xie , L. Zhang , et al., “Universal Soldering of Lithium and Sodium Alloys on Various Substrates for Batteries,” Advanced Energy Materials 8 (2017): 1701963.

[10] H. Fu , Q. Yin , Y. Huang , et al., “Reducing Interfacial Resistance by Na‐SiO2 Composite Anode for NASICON‐Based Solid‐State Sodium Battery,” ACS Materials Letters 2 (2019): 127–132.

[11] K. Lu , B. Li , X. Zhan , et al., “Elastic NaxMoS2‐Carbon‐BASE Triple Interface Direct Robust Solid‐Solid Interface for All‐Solid‐State Na‐S Batteries,” Nano Letters 20 (2020): 6837–6844. 32833461 10.1021/acs.nanolett.0c02871

[12] J. A. S. Oh , J. Sun , M. Goh , B. Chua , K. Zeng , and L. Lu , “A Robust Solid–Solid Interface Using Sodium–Tin Alloy Modified Metallic Sodium Anode Paving Way for All‐Solid‐State Battery,” Advanced Energy Materials 11 (2021): 2101228.

[13] Q. Zhang , Y. Lu , W. Guo , et al., “Hunting Sodium Dendrites in NASICON‐Based Solid‐State Electrolytes,” Energy Material Advances 2021 (2021): 1–10.

[14] C. Yuan , R. Li , X. Zhan , V. L. Sprenkle , and G. Li , “Stabilizing Metallic Na Anodes via Sodiophilicity Regulation: A Review,” Materials 15 (2022): 4636. 35806761 10.3390/ma15134636 PMC9267197

[15] Z. Yang , L. Chen , H. Jiang , et al., “SnF_2_‐Induced Highly Current‐Tolerant Solid Electrolytes for Solid‐State Sodium Batteries,” Advanced Functional Materials 33 (2023): 2306558.

[16] P. W. Jaschin , C. R. Tang , and E. D. Wachsman , “High‐Rate Cycling in 3D Dual‐Doped NASICON Architectures toward Room‐Temperature Sodium‐Metal‐Anode Solid‐State Batteries,” Energy & Environmental Science 14 (2024): 727–737.

[17] T. Liu , P. Xiang , Y. Li , et al., “In Situ Forming Na—Sn Alloy/Na2S Interface Layer for Ultrastable Solid State Sodium Batteries,” Advanced Functional Materials 34 (2024): 2316528.

[18] G. Deysher , J. A. S. Oh , Y.‐T. Chen , et al., “Design Principles for Enabling an Anode‐Free Sodium All‐Solid‐State Battery,” Nature Energy 9 (2024): 1161–1172.

[19] T. R. Jow and C. C. Liang , “Interface between Solid Anode and Solid Electrolyte‐Effect of Pressure on Li‐LiI(Al2O3) Interface,” Solid State Ionics 9‐10 (1983): 695–698.

[20] R. C. Galloway and S. Haslam , “The ZEBRA Electric Vehicle Battery‐ Power and Energy Improvements,” Journal of Power Sources 80 (1999): 164–170.

[21] T. Oshima , M. Kajita , and A. Okuno , “Development of Sodium‐Sulfur Batteries,” International Journal of Applied Ceramic Technology 1 (2004): 269–276.

[22] K. B. Hueso , M. Armand , and T. Rojo , “High Temperature Sodium Batteries: Status, Challenges and Future Trends,” Energy & Environmental Science 6 (2013): 734–749.

[23] G. S. Li , X. C. Lu , J. Y. Kim , et al., “Advanced Intermediate Temperature Sodium‐Nickel Chloride Batteries with Ultra‐High Energy Density,” Nature Communications 7 (2016): 10683. 10.1038/ncomms10683 PMC4753253 26864635

[24] X. C. Lu , G. S. Li , J. Y. Kim , et al., “Liquid‐Metal Electrode to Enable Ultra‐Low Temperature Sodium‐Beta Alumina Batteries for Renewable Energy Storage,” Nature Communications 5 (2014): 4578. 10.1038/ncomms5578 25081362

[25] M. M. Gross , L. J. Small , A. S. Peretti , S. J. Percival , M. A. Rodriguez , and E. D. Spoerke , “Tin‐Based Ionic Chaperone Phases to Improve Low Temperature Molten Sodium‐NaSICON Interfaces,” Journal of Materials Chemistry A 8 (2020): 17012–17018.

[26] Y. Cheng , M. Li , X. Yang , et al., “Na‐K Alloy Anode for High‐Performance Solid‐State Sodium Metal Batteries,” Nano Letters 22 (2022): 9614–9620. 36454039 10.1021/acs.nanolett.2c03718

[27] Y. Li , Z. Wang , C. Sun , et al., “Na‐K Interlayer Driven Na‐NASICON Solid‐State Batteries,” Advanced Functional Materials. (2025): 2425995.

[28] T. Yang , S. Qin , S. Gao , et al., “Electroinitiated Interfacial Healing for External Pressure‐Free Solid‐State Sodium Metal Batteries,” Nature Communications 16 (2025): 9613. 10.1038/s41467-025-64612-7 PMC12575646 41168187

[29] H. Liu , X.‐B. Cheng , J.‐Q. Huang , et al., “Alloy Anodes for Rechargeable Alkali‐Metal Batteries: Progress and Challenge,” ACS Materials Letters 1 (2019): 217–229.

[30] D. Reed , G. Coffey , E. Mast , et al., “Wetting of Sodium on β’’‐Al2O3/YSZ Composites for Low Temperature Planar Sodium‐Metal Halide Batteries,” Journal of Power Sources 227 (2013): 94–100.

[31] H.‐J. Chang , X. Lu , J. F. Bonnett , et al., “Decorating β″‐Alumina Solid‐State Electrolytes with Micron Pb Spherical Particles for Improving Na Wettability at Lower Temperatures,” Journal of Materials Chemistry A 6 (2018): 19703–19711.

[32] M. Y. M. Li , X. C. Lu , X. W. Zhan , et al., “High Performance Sodium‐Sulfur Batteries at Low Temperature Enabled by Superior Molten Na Wettability,” Chemical Communications 57 (2021): 45–48. 33325930 10.1039/d0cc06987f

[33] M. M. Li , S. Tripathi , E. Polikarpov , et al., “Interfacial Engineering with a Nanoparticle‐Decorated Porous Carbon Structure on Beta’’‐Alumina Solid‐State Electrolytes for Molten Sodium Batteries,” ACS Applied Materials & Interfaces 14 (2022): 25534–25544. 35608361 10.1021/acsami.2c05245

[34] D. Jin , S. Choi , W. Jang , et al., “Bismuth Islands for Low‐Temperature Sodium‐Beta Alumina Batteries,” ACS Applied Materials & Interfaces 11 (2019): 2917–2924. 30580514 10.1021/acsami.8b13954

[35] J. Brehm , M. Altmann , L. Mehlsam , P. Kotter , and A. Jossen , “Temperature Dependency of Swelling Force in Prismatic Lithium‐Ion Cells Under Fixed Mechanical Constraints Over Lifetime,” Journal of the Electrochemical Society 172 (2025): 070506.

[36] H. J. Chang , X. C. Lu , J. F. Bonnett , et al., “Development of Intermediate Temperature Sodium Nickel Chloride Rechargeable Batteries Using Conventional Polymer Sealing Technologies,” Journal of Power Sources 348 (2017): 150–157.

[37] A. C. Baclig , G. McConohy , A. Poletayev , et al., “High‐Voltage, Room‐Temperature Liquid Metal Flow Battery Enabled by Na‐K|K‐β″‐Alumina Stability,” Joule 2 (2018): 1287–1296.

[38] J. Mark Weller , H. H. Han , E. Polikarpov , et al., “Intrinsically Sodiophilic, Mesoporous Metal‐Free Wetting Layers Based on Inexpensive Carbon Black for Sodium‐Metal Batteries,” Nano Energy 128 (2024): 109815.

[39] D. Landmann , G. Graeber , M. V. F. Heinz , S. Haussener , and C. Battaglia , “Sodium Plating and Stripping from Na‐β"‐Alumina Ceramics beyond 1000 mA/cm2,” Materials Today Energy 18 (2020): 100515.

[40] A. S. Peretti , E. D. Spoerke , M. E. Ureña , et al., “Machinable, High‐Conductivity NaSICON through Mitigation of Humidity Effects during Solid‐State Synthesis,” Journal of the American Ceramic Society 109 (2025): e70195.

[41] M. J. Wang , R. Choudhury , and J. Sakamoto , “Characterizing the Li‐Solid‐Electrolyte Interface Dynamics as a Function of Stack Pressure and Current Density,” Joule 3 (2019): 2165–2178.

[42] T. Krauskopf , H. Hartmann , W. G. Zeier , and J. Janek , “Toward a Fundamental Understanding of the Lithium Metal Anode in Solid‐State Batteries‐An Electrochemo‐Mechanical Study on the Garnet‐Type Solid Electrolyte Li(6.25)Al(0.25)La(3)Zr(2)O(12),” ACS Applied Materials & Interfaces 11 (2019): 14463–14477. 30892861 10.1021/acsami.9b02537

[43] H. Koshikawa , S. Matsuda , K. Kamiya , et al., “Dynamic Changes in Charge‐Transfer Resistance at Li Metal/Li7La3Zr2O12 Interfaces during Electrochemical Li Dissolution/Deposition Cycles,” Journal of Power Sources 376 (2018): 147–151.

[44] B. Xiao , X. Liu , M. Song , et al., “A General Strategy for Batch Development of High‐Performance and Cost‐Effective Sodium Layered Cathodes,” Nano Energy 89 (2021): 106371.

[45] T. Deng , X. Ji , L. Zou , et al., “Interfacial‐Engineering‐Enabled Practical Low‐Temperature Sodium Metal Battery,” Nature Nanotechnology 17 (2022): 269–277. 10.1038/s41565-021-01036-6 34949775

[46] Q. Ma , T. Ortmann , A. Yang , et al., “Enhancing the Dendrite Tolerance of NaSICON Electrolytes by Suppressing Edge Growth of Na Electrode along Ceramic Surface,” Advanced Energy Materials 12 (2022): 2201680.

[47] J. Suo , Q. Zhao , H. Tian , et al., “Designing a Quasi‐Liquid Alloy Interface for Solid Na‐Ion Battery,” ACS Nano 17 (2023): 10229–10235. 37205737 10.1021/acsnano.3c00397

[48] H. H. Han , J. M. Weller , J. P. Quinn , et al., “Designing Carbon Wetting Layers with Inorganic Salts as Additives for Enhanced Adhesion and Molten Sodium Wettability in Electrochemical Application,” Frontiers in Batteries and Electrochemistry 5 (2026): 1759353.

[49] K. Jung , H.‐J. Chang , J. F. Bonnett , N. L. Canfield , V. L. Sprenkle , and G. Li , “An Advanced Na‐NiCl2 Battery Using Bi‐Layer (dense/Micro‐Porous) β″‐Alumina Solid‐State Electrolytes,” Journal of Power Sources 396 (2018): 297–303.

