# Supplementary material for: A Dendrite‐Resistant Sodium/Porous‐Carbon Anode for Solid‐State Batteries: Strategies and Challenges for Low‐Pressure Operation
Source: ChemSusChem. 2026 Jul 15;19(14):e70879. doi: 10.1002/cssc.70879 (PMC13372236; doi:10.1002/cssc.70879)
Supplement: Supplementary file 1 — The authors have cited additional references within the Supporting Information, which is accessible at (DOI). [file CSSC-19-e70879-s001.pdf]

A Dendrite-Resistant Sodium/Porous-Carbon Anode for Solid-State Batteries – Strategies and Challenges for Low-Pressure Operation– Supporting Information

J. Mark Weller*[a], Joseph P. Quinn[a], Evgueni Polikarpov[a], Henry Hyungkyu Han[a], Marcos Lucero[a], and Guosheng Li*[a]

[a] - Battery Materials and Systems Group, Energy and Environment Directorate

Pacific Northwest National Laboratory

Richland, WA 99354, USA

Correspondence E-mail: mark.weller@pnnl.gov, guosheng.li@pnnl.gov

Estimation of Maximum Cell Pressure

To assemble symmetric coin cells, a pneumatic cell crimper was used, with a fixed crimping pressure of 65 PSI (~ 0.45 MPa). The piston used for pressing has a diameter of 20 mm, therefore an area of ~ 3.1416 cm^2, translating to a pressing force of 143.4 N. Based on the area of the BASE electrolyte disks (~ 2 cm^2), this force translates to a pressure of ~ 0.72 MPa on the active components of the cell.

Regardless of which spring type (wave vs. belleville) is used, this 0.72 MPa pressure on the Na-BASE-Na symmetric cell is the maximum possible pressure that could be present during or after crimping. The rationale for this conclusion is that, if the pressing force of the cell crimper was too low to deform the spring, the cell would not be able to be crimped. Therefore, after crimping, the actual force applied to the cell from the spring must be equal to or less than the 143.4 N crimping force. Noting this maximum, possible pressure, we believe the actual pressure state in the cell to be substantially lower – however quantitative determination is difficult, hence the qualitative use of a light or stiff spring to assess the effect of pressure on cell cycling.

Discussion of Qualitative Assessment of Cycling Performance

Several cells were tested under controlled conditions including temperature (25 and 60 °C), pressure (effectuated by stiff vs. light springs), and current density (1 vs. 3 mA cm^{-2}) with a fixed cycling capacity of 1 mAh cm^{-2}. The criteria for performance assessment are:

1 - # of cycles before polarization to +/- 1 V voltage cutoffs (i.e., inability to reach target 1 mAh cm^{-2} capacity)

2 – whether one or both electrodes exhibit polarization behavior

3 – whether the cell experience short circuit due to dendrite intrusion

These factors for Cells 5-12 are summarized in **Table S2** based on the voltage vs. time traces shown in **Figure 3c-j**.

The following ratings are used:

Great – the cell cycled with no polarization to the voltage cutoffs after more than 20 cycles for bother electrodes

Good – the cell experienced polarization to the voltage cutoffs after more than 20 cycles for one or both electrodes

Reasonable – the cell experienced polarization to the voltage cutoffs in less than 10 cycles for only one electrode

Shorted – the cell failed due to dendrite induced short circuit during testing

Supporting Tables

Table S1 – List of all cells used in this study

Cell #	Current	Capacity	Temperature	Pressure	CCD	Max
**1**	Up to 3 mA cm^{-2}	Up to 1.5 mAh cm^{-2}	20-60 °C	Low	n/a	
**2**	0.6 mA cm^{-2}	0.25 mAh cm^{-2}	25 °C	Low	0.6 mA cm^{-2}	
**3**	0.6 mA cm^{-2}	0.25 mAh cm^{-2}	60 °C	Low	n/a	
**4**	10 mA cm^{-2} / 5.5 mA cm^{-2}	0.25 mAh cm^{-2}/0.5 mAh cm^{-2}	25 °C	Low	> 10 mA cm^{-2}/N/A	
**5**	3 mA cm^{-2}	1 mAh cm^{-2}	60 °C	High	n/a	
**6**	1 mA cm^{-2}	1 mAh cm^{-2}	60 °C	High	n/a	
**7**	3 mA cm^{-2}	1 mAh cm^{-2}	25 °C	High	n/a	
**8**	1 mA cm^{-2}	1 mAh cm^{-2}	25 °C	High	n/a	
**9**	3 mA cm^{-2}	1 mAh cm^{-2}	60 °C	Low	n/a	
**10**	1 mA cm^{-2}	1 mAh cm^{-2}	60 °C	Low	n/a	
**11**	3 mA cm^{-2}	1 mAh cm^{-2}	25 °C	Low	3 mA cm^{-2}	
**12**	1 mA cm^{-2}	1 mAh cm^{-2}	25 °C	Low	n/a	
**13**	1 mA cm^{-2}	0.25 mAh cm^{-2}	25 °C	Low	n/a	
**14**	0.125, 0.25, and 0.5 mA cm^{-2}	Up to 1.75 mAh cm^{-2}	25 °C	Low	n/a**	

Table S2 – Comparison of performance parameters for all symmetric cells in test matrix (see **Figure 3**)

Cell #	Cycles Before Polarization	# of Electrodes Polarizing	Short Circuit Failure?	Overall Rating
5	69/ > 74	1	No	Great
6	95	0	No	Great
7	5/ > 30	1	No	Reasonable
8	2/ > 13	1	No	Reasonable
9	4/12	2	No	Reasonable
10	12/25	2	No	Good
11	3/6	2	Yes	Failure, Shorted
12	7/13	2	No	Reasonable

Table S3 – Circuit fit parameters for solid-state Na symmetric cell impedance spectra

Cell #	R1	R2	Q2	a2	R3	Q3	a3	R4	Q4	a4
**9 – Pre Cyc**	4.72	0.37	0.00016	0.75	1.34	3.58	0.54	-	-	-
**9 – Post Cyc**	5.89	8.31	2.28E-05	0.62	7.97	0.01	0.57	16.72	0.29	0.5
**10 – Pre Cyc**	4.82	0.19	0.00036	0.78	1.09	4.39	0.59	-	-	-
**10 – Post Cyc**	7.28	2.44	6.50E-07	0.89	5.39	0.09	0.33	19.13	0.53	0.45

R – unit of Ω

Q – unit of F s^{a-1}

a – unitless

Supporting Figures

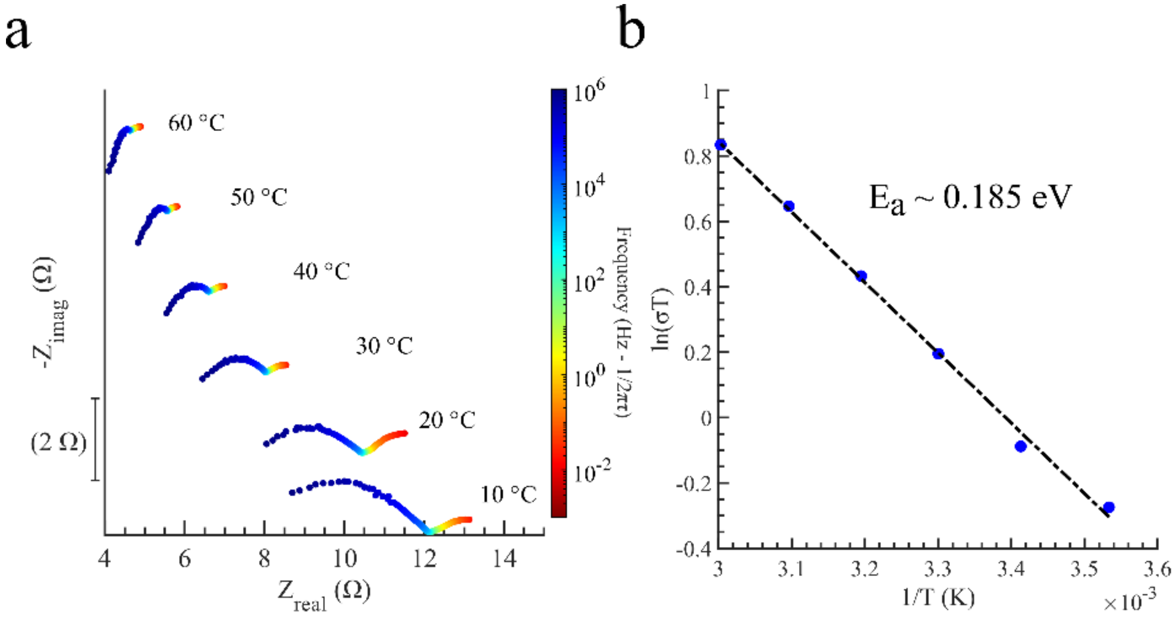

Figure S1 - electrochemical performance of Cell 1 showing a) EIS spectra at various temperatures and b) Arrhenius plot of ionic conductivity and determination of activation energy of Na-β"-Al_2O_3/Y-Stabilized ZrO_2 composite solid electrolyte

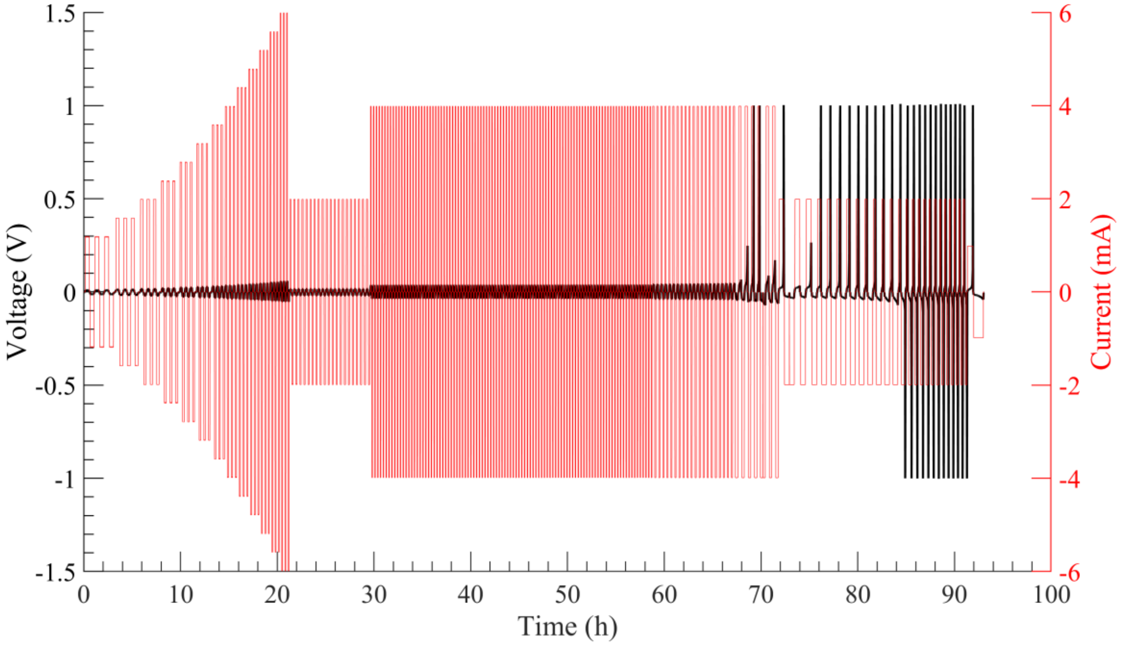

Figure S2 – full cycling history of Cell 1 shown in **Figure 1** at 50 °C.

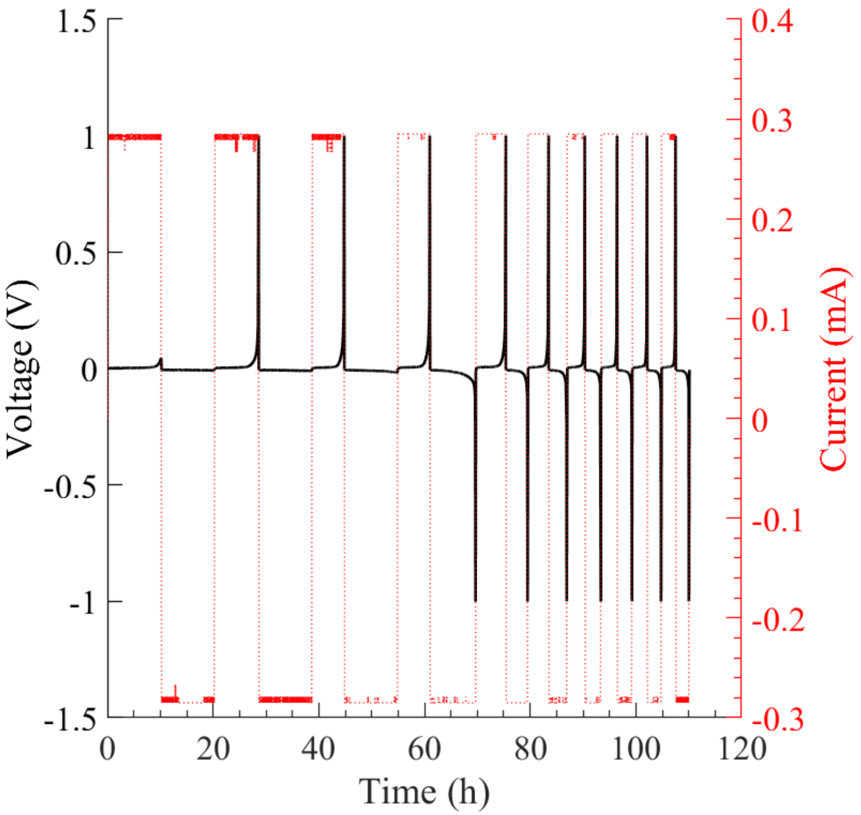

Figure S3 - Cell NaSSB-5 low current, high capacity cycling data at 60 °C revealing eventual polarization behavior even at low current density after initial successful plating and stripping of ~ 3 mAh capacity

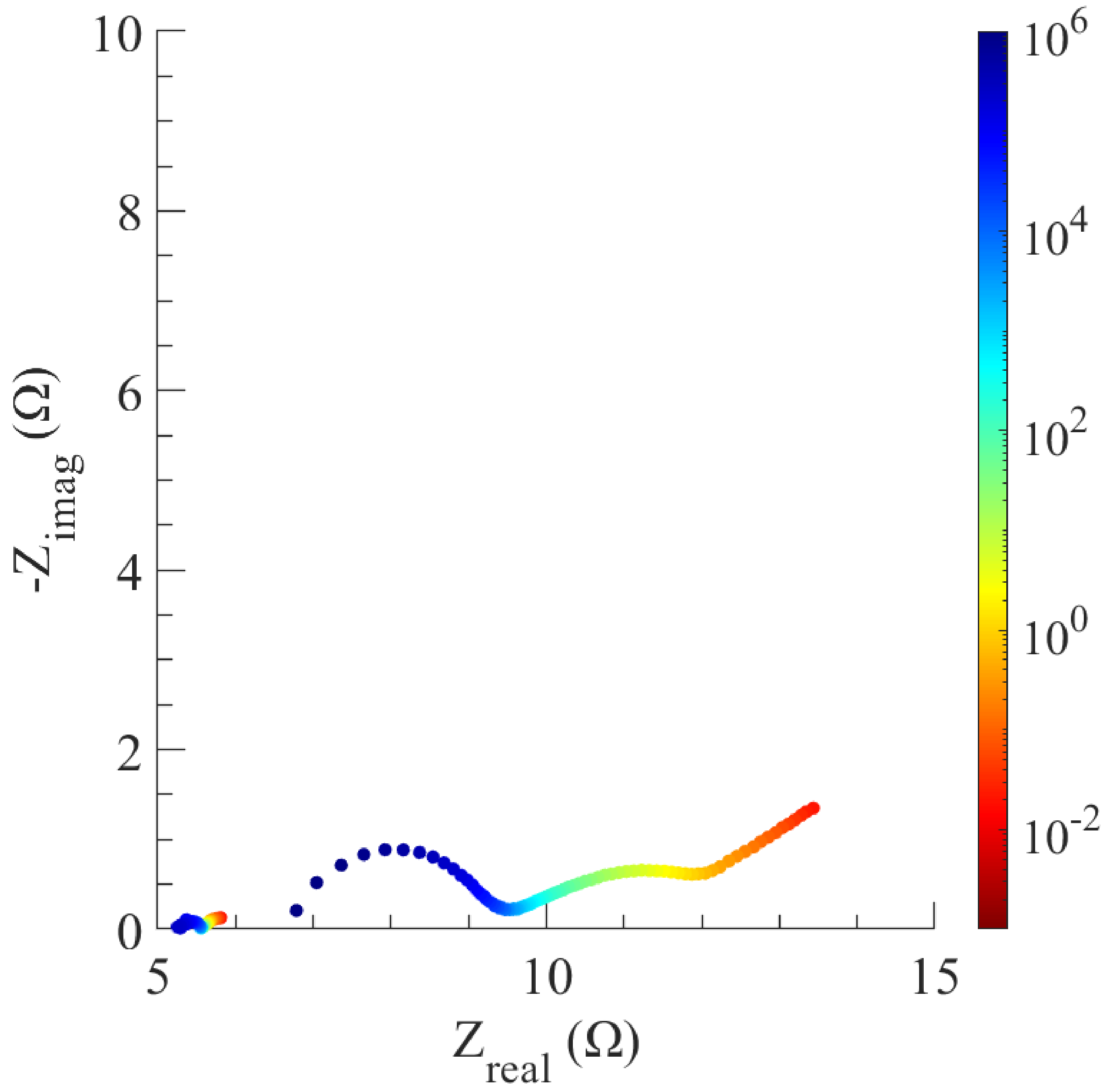

Figure S4 – EIS spectra of Cell Na-SSB 5 before (left) and after (right) cycling at 50 °C, showing significant increase in polarization and emergence of an intermediate frequency charge transfer response and more pronounced diffusion tail. Note that the datapoints are colored by frequency (Hz, see color bar on right).

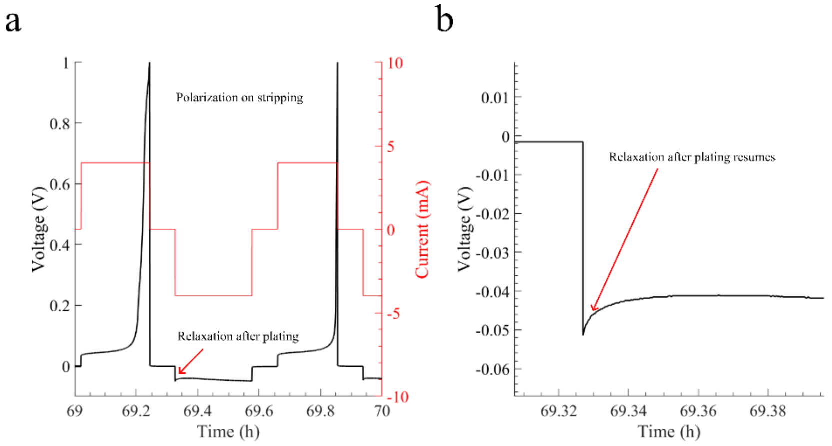

Figure S5 - Zoomed region of voltage trace of Cell Na-SSB 5 (**Figure S2**) showing **a)** zoomed region of overpolarization on ‘charge’ i.e. stripping from the ‘cathode’ side of the cell and subsequent relaxation after resuming plating on ‘discharge,’ with this effect highlighted in **b)** where a relaxation and equilibration of the voltage after initially reversing polarity can be clearly perceived.

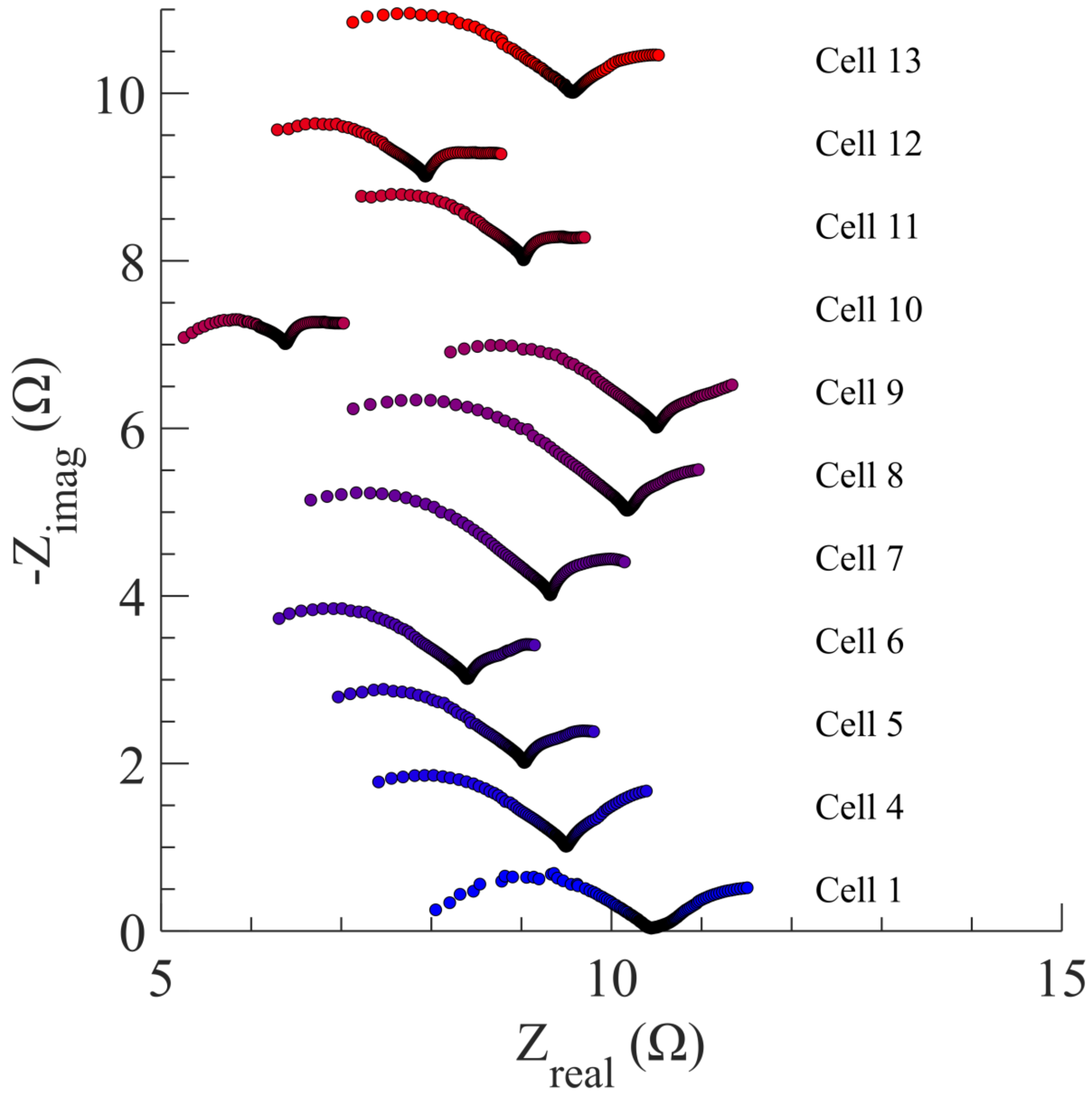

Figure S6 - all porous carbon-based Na-Na cells EIS comparison with variability related to slight differences in BASE thickness and coverage of porous carbon interlayer

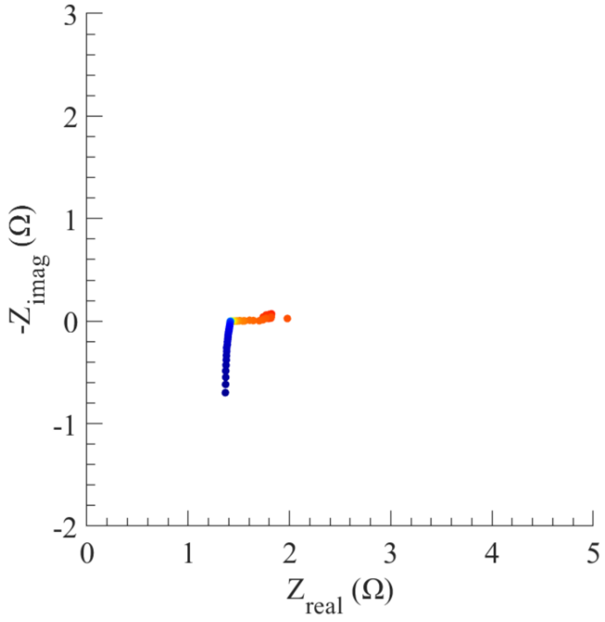

Figure S7 - post-cycling EIS spectrum of Cell 2 (corresponding to **Figure 2a**) after obvious dendrite induced short circuit

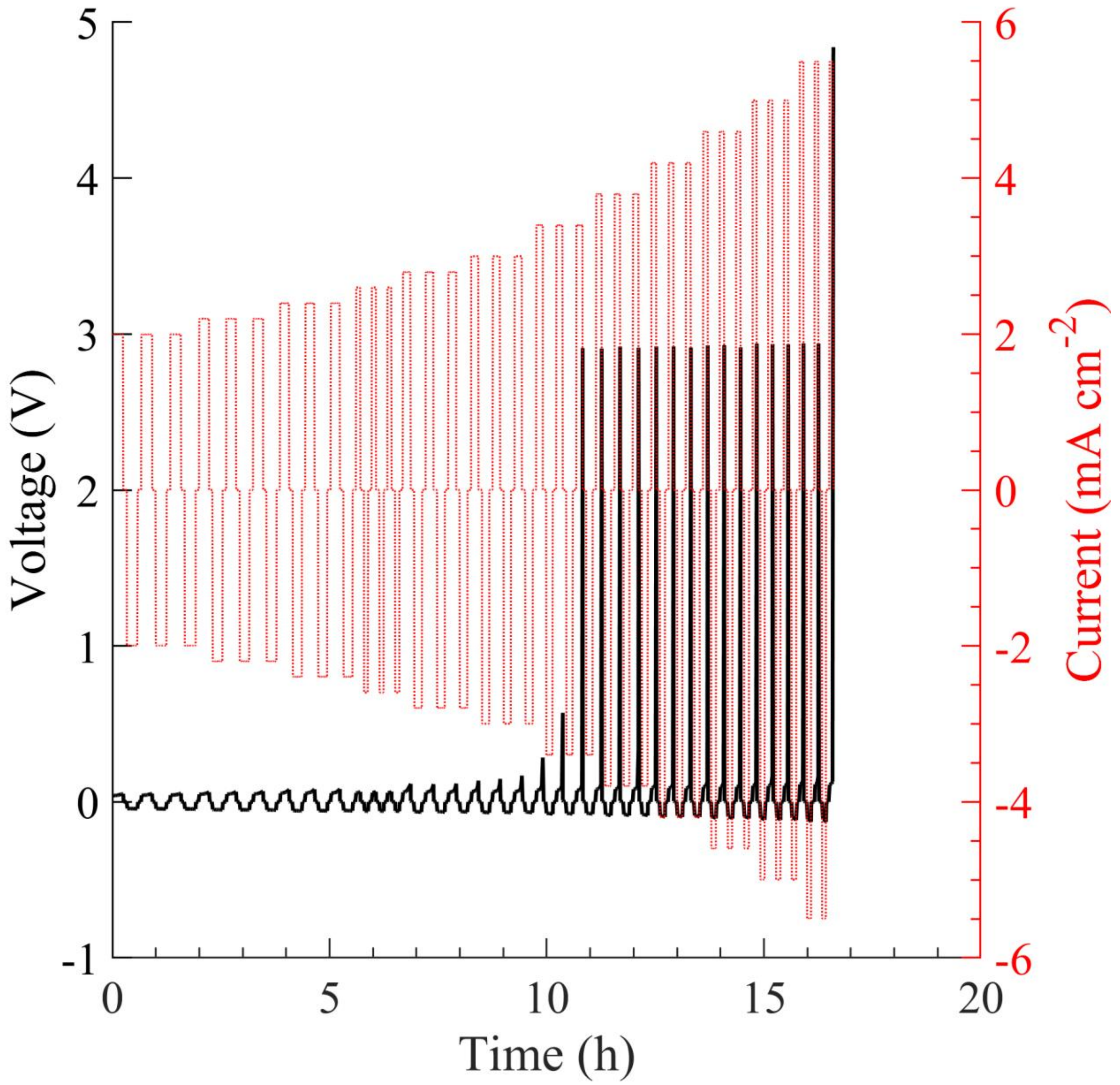

Figure S8 – voltage and current traces for Cell #4 with a fixed 0.5 mAh cm^{-2} cycling capacity

References

1 Lasia, A. *Electrochemical Impedance Spectroscopy and its Applications*. (Springer, 2014).
